# Comparative Analysis of Salivary Bacterial Microbiome Diversity in Edentulous Infants and Their Mothers or Primary Care Givers Using Pyrosequencing

**DOI:** 10.1371/journal.pone.0023503

**Published:** 2011-08-10

**Authors:** Kimberly D. Cephas, Juhee Kim, Rose Ann Mathai, Kathleen A. Barry, Scot E. Dowd, Brandon S. Meline, Kelly S. Swanson

**Affiliations:** 1 Division of Nutritional Sciences, University of Illinois, Urbana, Illinois, United States of America; 2 Department of Kinesiology and Community Health, University of Illinois, Urbana, Illinois, United States of America; 3 Department of Animal Sciences, University of Illinois, Urbana, Illinois, United States of America; 4 Research and Testing Laboratory and Medical Biofilm Research Institute, Lubbock, Texas, United States of America; 5 Department of Maternal and Child Health, Champaign-Urbana Public Health District, Champaign, Illinois, United States of America; Baylor College of Medicine, United States of America

## Abstract

Bacterial contribution to oral disease has been studied in young children, but there is a lack of data addressing the developmental perspective in edentulous infants. Our primary objectives were to use pyrosequencing to phylogenetically characterize the salivary bacterial microbiome of edentulous infants and to make comparisons against their mothers. Saliva samples were collected from 5 edentulous infants (mean age = 4.6±1.2 mo old) and their mothers or primary care givers (mean age = 30.8±9.5 y old). Salivary DNA was extracted, used to generate DNA amplicons of the V4–V6 hypervariable region of the bacterial 16S rDNA gene, and subjected to 454-pyrosequencing. On average, over 80,000 sequences per sample were generated. High bacterial diversity was noted in the saliva of adults [1012 operational taxonomical units (OTU) at 3% divergence] and infants (578 OTU at 3% divergence). Firmicutes, Proteobacteria, Actinobacteria, and Fusobacteria were predominant bacterial phyla present in all samples. A total of 397 bacterial genera were present in our dataset. Of the 28 genera different (P<0.05) between infants and adults, 27 had a greater prevalence in adults. The exception was *Streptococcus*, which was the predominant genera in infant saliva (62.2% in infants vs. 20.4% in adults; P<0.05). *Veillonella*, *Neisseria*, *Rothia*, *Haemophilus*, *Gemella*, *Granulicatella*, *Leptotrichia*, and *Fusobacterium* were also predominant genera in infant samples, while *Haemophilus, Neisseria, Veillonella, Fusobacterium, Oribacterium, Rothia, Treponema*, and *Actinomyces* were predominant in adults. Our data demonstrate that although the adult saliva bacterial microbiome had a greater OTU count than infants, a rich bacterial community exists in the infant oral cavity prior to tooth eruption. *Streptococcus, Veillonella,* and *Neisseria* are the predominant bacterial genera present in infants. Further research is required to characterize the development of oral microbiota early in life and identify environmental factors that impact colonization and oral and gastrointestinal disease risk.

## Introduction

Given the high dental caries prevalence in US pre-school children (28%) [1], a renewed focus on oral disease detection and prevention is needed. Oral disease is known to be of microbial origin, but research has been hampered by the methodology to study bacteria. Early experiments reliant on culture methods identified *Streptococcus mutans* to be a primary contributor to dental caries [2] and other species such as *Porphyromonas gingivalis*, *Tannerella forsythia*, and *Treponema denticola* to be contributors of periodontal diseases [3]. The mere presence of *S. mutans*, however, is often not reliable in predicting caries lesions [4], suggesting the presence of other microbial strains and environmental etiology such as food consumption patterns. Recent experiments using DNA-based methods have not only demonstrated that the majority of strains are not culturable and that the oral microbiome is far more diverse than originally thought, but that oral disease is polymicrobial in nature [5]–[7]. For example, several other taxa including *Actinomyces*, *Granulicatella*, *Veillonella*, *Bifidobacteriaceae*, and *Scardovia* have all been identified as contributors to early childhood caries recently [8]–[10]. Thus, the application of molecular techniques is critical in defining the unique microbial niches within the mouth (e.g., tongue, teeth, soft tissues) and identifying how these populations change with age or disease progression.

While many researchers have used molecular methods to study specific microbial species or families, others have focused on the host and have used cloning and traditional (Sanger) sequencing to characterize the oral microbiota of healthy and diseased patients, including children. Aas et al. [11] used sequencing analysis of 1,285 16S rRNA clones and a reverse capture checkerboard assay to determine bacterial species associated with oral health and dental caries of permanent teeth of children and young adults (2 to 21 y old). In their preliminary study of 42 subjects with severe dental caries, they noticed that plaque from approximately 10% had no detectable levels of *S. mutans*, suggesting an important role of other microbial pathogens. In a population of 93 children, Gizani et al. [12] compared microbial populations of five different intra-oral habitats (i.e., saliva, tongue dorsum, soft tissues, and supragingival and subgingival plaque) and across age groups (3 to 6 y old; 6 to 9 y old; 9 to 12 y old) using checkerboard DNA-DNA hybridization. Of importance, they reported that cariogenic bacteria, including *S. mutans*, *S. sobrinus*, and *L. acidophilus*, were present in nearly all children and locations sampled. Because the soft tissues, saliva, and tongue were more colonized with Streptococcal species than teeth, their importance as reservoirs for oral pathogens was highlighted. More recently, pyrosequencing and Illumina high-throughput sequencing, techniques that are much cheaper and faster than traditional sequencing, have been applied to oral microbe communities [6], [13], [14], providing even deeper characterization of the oral microbiome.

Despite the dramatic progress made as it pertains to the oral microbiome in recent years, a paucity of information exists in the edentulous infant. Although a high bacteria count has been reported in adult edentulous mouths (1.58×10^9^ bacteria) [15] and a rich microbial population is known to exist in the oral soft tissues of young children [12], little has been done in edentulous infants. Thus, our objectives in this experiment were to characterize the phylogeny of the infant salivary bacterial microbiome using bar-coded 454-pyrosequencing, and to assess the phylogenetic differences between infants and their mothers. Based on the current literature, we hypothesized that: 1) the infant salivary bacterial microbiome would be less diverse than that of adults (mothers or primary caregivers); 2) the salivary bacterial microbiome of each infant would be more similar to their own mother as compared to other mothers; and 3) that pathogens associated with dental disease were present in the oral cavity prior to tooth eruption.

## Results

Saliva samples were collected from 5 edentulous infants (4 males; 1 female) with a mean age = 4.6±1.2 mo old. Age, sex, feeding experiences, and oral hygiene practices were different among the infants studied and are provided in Table 1. Samples were also collected from adults providing the primary care to these infants. Four were biological mothers and one was a biological grandmother (mean age = 30.8±9.5 y old). Self-reported ethnicities of study participants were White (2 infants and 2 adults) and African-American (3 infants and 3 adults), who were all enrolled in the Women, Infants and Children (WIC) Program at the Champaign-Urbana Public Health District. Due to poor quality and quantity of DNA, the sample from one mother was not subjected to pyrosequencing, leaving 5 infant and 4 adult samples for analyses.

**Table 1:** Age, sex, feeding experiences and oral hygiene practices of babies in study.

Item	Baby 1	Baby 2	Baby 4	Baby 6	Baby 8
Age (mo)	6	3	4	4	6
Sex	Male	Male	Female	Male	Male
Feeding experiences					
Ever breastfed?	Yes	No	Yes	No	Yes
Breastfeeding duration	3 months	None	1 day	None	6 months
Time of formula introduction	Day 1	Day 1	Day 1	Day 1	Day 7
Solid foods introduced?	Yes	No	Yes	Yes	No
Foods offered	Cereal; baby fruits & vegetables; juice	None	Cereal; baby fruits & vegetables; juice	Teething biscuits	None
Oral hygiene practices used					
Oral hygiene practices	Baby finger brush; fluorinated water	Wipe out (cloth); rinse with water	Baby finger brush; bottled water	Wipe out (cloth)	Baby finger brush
Time last cleaned baby's mouth (hr)	0.5	4	19	5	24
Time elapsed since last meal (min)	120	60	15	30	5

We generated an average of over 80,000 sequences from each infant and adult sample that were utilized for analysis (Table 2). Sequences are available at the NCBI sequence read archive (http://www.ncbi.nlm.nih.gov/Traces/sra/) under accession number SRP006873. After trimming the primer sequences, the average sequence length was approximately 516 and 513 nt for infants and adults, respectively. Bacterial diversity was assessed using Mothur and QIIME [16], [17], as these two methods are well documented approaches at evaluating alpha diversity**.** Because our dataset only included 5 babies and 4 adults, it is difficult to adequately test the salivary bacterial microbiome of this population. However, Mothur identified a higher number of operational taxonomical units (OTU) for adults (mean OTU at 3% dissimilarity = 1,012) than infants (mean OTU at 3% dissimilarity = 578; Table 2). QIIME utilized final sequence counts of 36,422 for infants and 30,377 which were then analyzed to generate maximum observed for adults averaging 676 OTU at 3% divergence for infants and 895 OTU for adults. Both methods utilized here failed to reach a plateau of the rarefaction curves (maximum OTU) although the numbers for QIIME and the High Quality sequences method utilized with Mothur generated similar estimates. To predict the maximum OTU based upon the rarefaction curves, a Richard's equation was utilized. The maximum predicted for infants and adults using QIIME data was 1,417 and 1,645. Similar values were found using Mothur approach with the normalized set of high quality sequences was 1,151 for infants and 2,264 for adults.

**Table 2:** Number of sequences obtained from and similarity-based species richness estimates obtained from 4,600-sequence subsamples using high quality sequence MOTHUR with maximum predicted OTU based upon Richard's equation and QIIME.

		Parameters calculated using 4,600-sequence subsamples		
Sample	Original Sequences^1^	oOTU^2^	mpOTU^3^	qOTU^4^
Baby 1	104,727 (50,989)	860	1670	2008
Baby 2	81,102 (38,824)	638	1313	1278
Baby 4	81,078 (39,915)	432	867	1411
Baby 6	114,638 (56,145)	747	1481	1887
Baby 8	43,694 (27,191)	215	426	500
Mother 1	127,306 (62,670)	1103	2356	1958
Mother 2	118,746 (56,732)	1515	3892	2317
Mother 6	43,040 (20,992)	815	1515	1251
Mother 8	41,468 (20,726)	616	1295	1053
Mean baby^5^	85,048 (42,612)	578±257.3	1151±502.7	1417±598.0
Mean mother^5^	82,640 (40,280)	1012±390.3	2264±1177.4	1645±593.1

1 Total number of sequences utilized for taxonomic analysis (total number of reverse sequences) Mean for baby and mothers based upon reverse sequence reads which were utilized for rarefaction analysis and Unifrac-based analysis.

2 Operational Taxonomical Unit at 3% dissimilarity based upon high quality sequence selected MOTHUR (HQSM) observed rarefaction.

3 Operational Taxonomical Unit at 3% dissimilarity based upon MOTHUR rarefaction with Richard's equation prediction of maximum OTU.

4 Operational Taxonomical Unit at 3% dissimilarity based upon QIIME observed rarefaction with Richard's equation predicted maximum OTU.

5 Baby and mother oOTU, mpOTU, and qOTU means±standard deviation.

Based upon these estimates, however, there was a significant (P<0.01) correlation or interaction detected in relation to the number of sequences within each sample on the diversity estimates when using the QIIME approach. Thus, we might assume that because the sequence numbers for the adults was much lower than that of the infants, that the QIIME based estimates for the diversity in adults may be underestimated slightly. In two instances with QIIME, the infant-adult paired samples showed higher diversity in the infants which was in turn correlated with an increased number of sequences evaluated. Interestingly, with a force to nearest species approach using BLASTn, infants resolved into 620 unique species designations while adults resolved to 734 which was very similar to the QIIME results (data not shown).

The diversity estimates based upon the normalized high quality sequence Mothur approach (HQSM) were not able to be evaluated in this manner, as all samples were evaluated with similar numbers of sequences (approximately 4,600 each). Based upon the data generated using the mothur approach, the primary hypothesis that the infants have lower diversity than the adults appeared to be correct, as in all instances [based upon both the predicted maximum (Richard's model) and observed OTU for infants] tended to be lower (P<0.10) than that of adults. Our sample number, however, provided low statistical power. More research studying a larger population of infants is required to adequately test this hypothesis.

**Figure 1:**
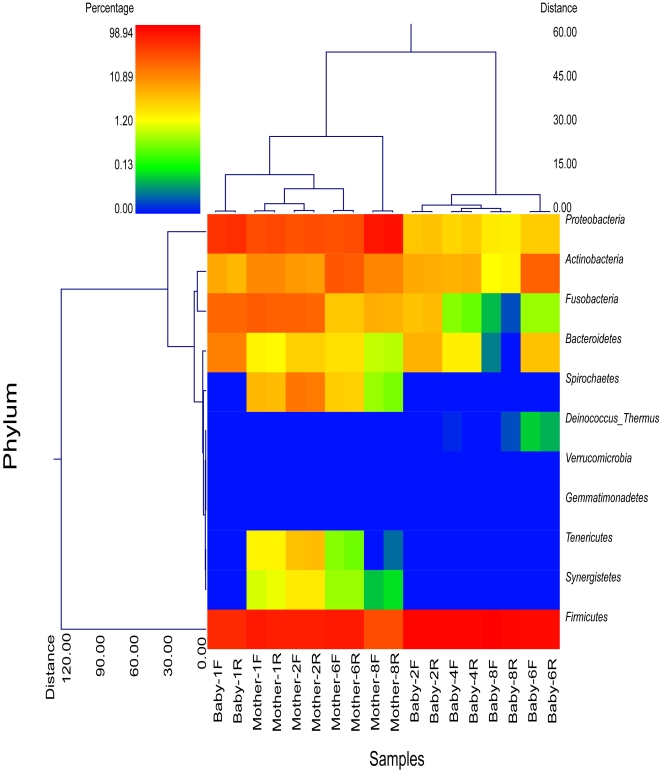
Dual hierarchical clustering dendogram of the bacterial phyla profiles for babies and mothers. Baby and mother number and sequence direction [forward (F); reverse(R)] are provided on the bottom x-axis. The dendogram provides the phylum designation along the right y-axis and the abundance relationship across all samples for each phylum based upon Wards clustering and Manhattan distance methods along the left y-axis. The relative distance scale for the left y-axis is provided in the lower left corner of the figure. The samples have also been clustered based upon Wards and Manhattan principles with sample designations along the bottom x-axis and the clustering described along the top x-axis in the figure. The heatmap depicts the relative percentage of each phyla for each sample. The color scale for the heatmap is shown in the upper left corner of the figure. It is obvious from the figure that the microbiome of the mothers and babies are clearly separable but forward and reverse reads cluster together.

Clustering based upon the phyla indicates that the forward and reverse reads from each sample are nearly identical (Figure 1), indicating that either orientation or reading from a single direction is acceptable for evaluating bacterial diversity and further validating the selection of reverse (1100R) sequences for diversity estimates with HQSM. Figure 1 also shows that infants, except for Baby 1, separate distinctly from the adults. Baby 1, who was the oldest infant and had been eating solid foods for a couple of months prior to saliva collection, was more closely related to the adults than to the other infants. Our hypothesis that an infant's salivary bacterial microbiome would be more similar to their own mother as compared to other mothers was not found to be true. The sample distance matrix between babies and their mothers (0.91±0.01) was similar to that of babies and unrelated mothers (0.92±0.02).

Despite our low sample number, several phyla were present in different (P<0.02) amounts between adults and infants, including Firmicutes, Synergistetes, Proteobacteria, Tenericutes, and Spirochetes. The predominant phyla present in saliva samples, representing approximately 95% of the sequences in both age groups, included Firmicutes (infants: 82.7±20.84%; adults: 48.5±19.19%), Proteobacteria (infants: 8.3±14.29%; adults: 32.3±22.64%), Actinobacteria (infants: 4.0±4.25%; adults: 7.6±4.38%), and Fusobacteria (infants: 2.8±4.64%; adults: 7.4±5.43%). Spirochaetes were only present in one infant at <0.002%, but were present in all of the adult samples (2.8±3.00%). Similarly, Synergistetes were absent from all infant samples but were present in all adult samples (0.3±0.25%) and likewise Tenericutes were absent from infant samples but present in all but one of the adult samples (0.6±0.63%). Finally, Bacteroidetes were more abundant in infant samples compared to adult samples (infants: 2.2±2.40%; adults: 0.65±0.58%) and Deinococcus-Thermus were present in a few of the infant samples but not found in adult samples.

A total of 397 bacterial genera and 1,033 species were identified in our dataset (Table S1 and S2, respectively). There were notable differences in genera between infants and adults (Table 3). *Streptococcus* was by far the genera of highest prevalence in infant saliva, accounting for 62.2% of all sequences and being present in greater (P<0.001) average abundance than in adults (20.4%). Twenty three genera accounted for 99% of diversity in infants, while 45 genera accounted for 99% in mothers. *Streptococcus* has species that are both associated with periodontal disease and health. However, all of the genera found to be significantly different between adults and infants have also been implicated in the association with periodontal disease [18]–[20]. All of these genera, with the exception of *Streptococcus,* were more abundant in adults.

**Table 3:** Bacterial genera present in different (P<0.05) amounts in baby vs. mother or primary care giver saliva samples.

	Babies				Mothers/Care Givers			
Genera	Min^1^	Max	Mean	SD	Min	Max	Mean	SD
*Streptococcus*	24.2	97.50	62.21	23.69	7.19	36.08	20.39	11.11
*Haemophilus*	0.03	11.10	2.63	4.31	2.11	25.72	16.41	9.07
*Fusobacterium*	0.00	6.03	1.25	2.22	1.37	12.86	6.49	4.84
*Actinomyces*	0.23	0.81	0.44	0.19	0.36	5.70	2.76	1.88
*Oribacterium*	0.00	1.69	0.38	0.64	1.47	13.72	6.30	4.83
*Campylobacter*	0.00	0.48	0.25	0.14	0.35	4.66	2.43	1.84
*Eubacterium*	0.00	0.70	0.21	0.25	0.54	2.09	1.34	0.62
*Megasphaera*	0.00	0.24	0.10	0.10	0.00	2.15	0.80	0.80
*Atopobium*	0.01	0.29	0.09	0.09	0.01	1.36	0.61	0.47
*Terrahaemophilus*	0.00	0.30	0.04	0.09	0.63	11.96	6.45	4.15
*Actinobacillus*	0.00	0.18	0.03	0.06	0.05	0.20	0.11	0.05
*Catonella*	0.00	0.10	0.02	0.04	0.03	1.25	0.43	0.42
*Kingella*	0.00	0.08	0.02	0.02	0.02	0.64	0.20	0.25
*Lachnospira*	0.00	0.05	0.01	0.02	0.01	1.15	0.31	0.36
*Mogibacterium*	0.00	0.05	0.01	0.02	0.03	0.66	0.21	0.26
*Corynebacterium*	0.00	0.03	0.01	0.01	0.04	0.30	0.14	0.09
*Peptostreptococcus*	0.00	0.03	0.01	0.01	0.09	0.62	0.24	0.18
*Selenomonas*	0.00	0.02	0.01	0.01	0.01	2.44	0.77	0.96
*Aggregatibacter*	0.00	0.01	0.00	0.03	0.14	1.47	0.55	0.47
*Filifactor*	0.00	0.02	0.00	0.01	0.08	3.01	0.78	1.01
*Dialister*	0.00	0.02	0.00	0.01	0.01	0.91	0.39	0.35
*Treponema*	0.00	0.00	0.00	0.00	0.10	8.23	2.76	3.03
*Lautropia*	0.00	0.00	0.00	0.00	0.01	1.15	0.41	0.41
*Aminobacterium*	0.00	0.00	0.00	0.00	0.01	0.45	0.16	0.14
*Bulleidia*	0.00	0.00	0.00	0.00	0.02	0.38	0.16	0.14
*Mycoplasma*	0.00	0.00	0.00	0.00	0.00	0.90	0.36	0.35
*Parvimonas*	0.00	0.00	0.00	0.00	0.01	1.12	0.38	0.41

1 Min – minimum; Max = maximum; SD = standard deviation.

**Figure 2:**
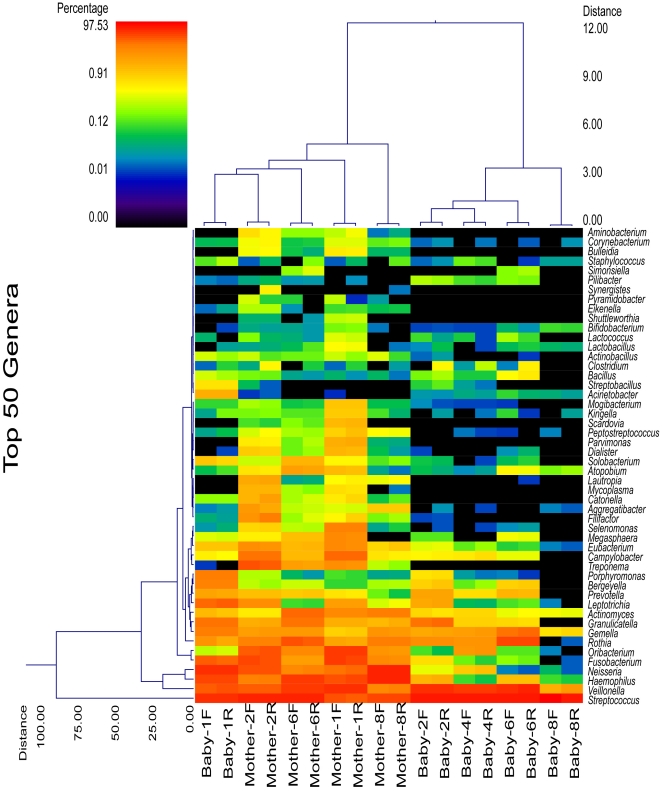
Dual hierarchical clustering dendogram of the most predominant and ubiquitous 50 bacterial genera among the samples. Baby and mother number and sequence direction [forward (F); reverse(R)] are provided on the bottom x-axis. Those genera that occurred in only 1 sample were omitted, along with those present in relative average percentages<0.09% in 3 or fewer samples. The genera that occurred in average percentages <0.01% among all samples were also excluded. It should be noted that inclusion of all genera did not change the clustering patterns (data not shown), thus this analysis accurately represents and overview and relationship among samples for the microbiome data. The dendogram provides the genus designation along the right y axis and the abundance relationship across all samples for each genus based upon Wards clustering and Manhattan distance methods along the left y-axis. The relative distance scale for the left y-axis is provided in the lower left corner of the figure. The samples have also been clustered based upon Wards and Manhattan principles with sample designations along the bottom x-axis and the clustering described along the top x-axis in the figure. The heatmap depicts the relative percentage of each genus for each sample. The color scale for the heatmap is shown in the upper left corner of the figure.

**Figure 3:**
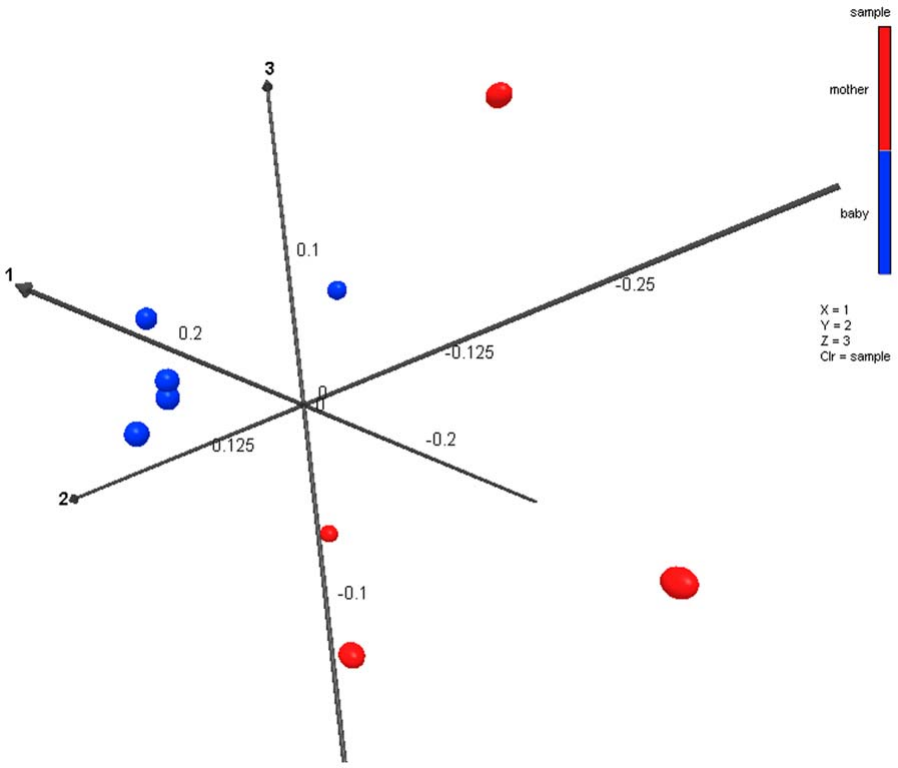
Principal component analysis of UNIFRAC distance metric. This figure provides a 3 dimensional visualization of the PCA analysis using the top 3 vectors. Red balls representing the *mother* samples and blue balls representing the baby samples clearly show the significant clustering distance between the two groups. Eigenvalues and % variation explained by each vector are Vector 1 eigval = 0.211 (41.5%), Vector 2 = 0.137 (26.8%), and Vector 3 0.061 (12%).

Wards clustering based upon Manhattan distance methods at the genus level, similar to clustering of phyla, shows that infants (except for Baby 1) clustered apart from adults when considering all genera (data not shown). Dual dendrogram of the top 50 most predominant genera among both adults and infants shows the same pattern (Figure 2). Both forward and reverse sequencing reads clustered each of the individual samples together. Further evaluation was also conducted using Unifrac based methods. These analyses also showed that adults grouped separately from infants, which was based upon principal component analysis (Figure 3) when considering the primary 3 vectors, which together accounted for 80.3% of the variation, and described a significant difference between infants and adults (P = 0.013).

## Discussion

Given their lack of teeth, infants have not been a population commonly studied as it pertains to oral microbial establishment and disease development. However, a description of the oral microbiota prior to tooth eruption may provide important information as to the evolution of the oral microbial community early in life and contribute to what is already known about the many factors (e.g., eating habits, dental health practices, etc.) that influence oral microbes and consequent disease incidence. Recently, researchers have examined oral microbe populations in young (3–12 yr old) children [7]–[12]. Results from these studies have suggested that the entire population of oral bacteria, rather than the presence of a small number of specific pathogens, have the greatest impact on disease risk.

Aas et al. [11] not only demonstrated that 10% of children and young adults (2 to 21 yr old) with dental caries had no detectable levels of *S. mutans*, but suggested the involvement of *Veillonella*, *Lactobacillus*, *Bifidobacterium*, *Propionibacterium*, low-pH non-*S. mutans* Streptococci, *Actinomyces*, and *Atopobium* in the development and progression of dental caries. *Actinomyces*, *Granulicatella*, *Veillonella*, *Bifidobacteriaceae*, and *Scardovia* have been identified as contributors to early childhood caries by others as well [8]–[10]. A study performed by Gizani et al. [12] demonstrated that cariogenic bacteria, including *S. mutans*, *S. sobrinus*, and *L. acidophilus*, were present in healthy and diseased children (3 to 12 yr old) and in all locations (saliva, tongue dorsum, soft tissues, and supragingival and subgingival plaque) sampled. Finally, Ling et al. [7] used PCR-DGGE and high-throughput sequencing to examine salivary and supragingival plaque microbes in 60 children (3 to 6 yr old) with or without dental caries from China. They demonstrated that the oral microbiota of children has much greater diversity than previously thought (>200 genera identified) and that no specific pathogen or pathogens (e.g., core microbiome), but pathogenic populations as a whole, were significantly associated with dental caries. Thus, using molecular assays that enable the study of the entire microbial population in a given environment is warranted.

To our knowledge, the recent study comparing newborn vs. mother oral, fecal, vaginal, and skin bacterial populations immediately after birth [21] and the current study are the only to report the use of high-throughput sequencing to examine the oral microbiome in edentulous infants <6 months of age. Bacterial diversity observed in our infant sample population was highly variable (426 to 1670 maximum predicted OTU) and much greater than anticipated, but not as great as that of mothers (1295 to 3892 OTU). We expected to observe a strong “legacy effect” (e.g., influence of a local environmental bacterial community or inheritance of bacteria from a parent) on the salivary bacterial microbiome of our infants, with infant populations being most similar to their own mother. The data from our study population, however, demonstrated that the oral bacteria of infants were no more similar to their own mothers than unrelated adults. Even though the PCA cluster analysis and dendrograms demonstrate that infant and mother microbiomes tended to cluster in two distinct groups, it was not the clear separation expected. Thus, even though our infant population did not yet have teeth, the environmental exposures during the first few months of life (e.g., diet, living conditions, siblings, pets, etc) had already dramatically altered the oral bacterial ecology.

At the phyla level, our results are in agreement with previous high-throughput sequencing studies of the oral microbiome in children [7] and adults [13], [22], with over 99% of all sequences being members of the following 7 phyla: Firmicutes (67.5%), Proteobacteria (18.9%), Actinobacteria (5.6%), Fusobacteria (4.8%), Spirochaetes (1.2%), and Bacteroidetes (1.5%). The primary difference between our study and that of Keijser et al. [22] and Ling et al. [7] was the prevalence of Bacteroidetes, which was second highest phyla behind Firmicutes in those studies, but was present in an average relative percentage <2% among our infant and adult samples. In agreement with the current study, Lazarevic et al. [13] also reported a lower prevalence to Bacteroidetes (<0.1%) in their dataset. We identified 303 genera in the adult saliva samples. Similar to that of recent experiments, predominance of *Streptococcus*, *Haemophilus*, *Veillonella*, *Neisseria*, *Fusobacterium*, *Oribacterium*, *Gemella, Granulicatella*, *Rothia*, *Actinomyces*, *Treponema*, *Campylobacter*, and *Leptotrichia* were observed, with all present at average levels >1% of sequences. The prevalence of the genera *Porphyromonas* and *Prevotella* was lower than that reported by other researchers [5], [6], [14], [22]. Lazarevic et al. [13], who used Illumina technology, reported a much less diverse genera population, with >70% belonging to *Streptococcus* or *Neisseria*. Primer bias, hypervariable region or length of the 16S gene product analyzed, human subject (biological) variation, and sample preparation (laboratory) differences among studies may contribute to these discrepancies.

We identified approximately 100 more bacterial genera in our infant saliva samples (261 genera) as that reported by Ling et al. [7] (i.e., 156 genera). This may have been affected by the hypervariable region of the 16S gene examined (V4–V6 vs. V3) or the sequence length examined (∼414 bp vs. ∼145 bp), but was likely due to the greater sequencing depth in our study (>80,000 vs.∼1,560 sequences/sample) which is known to influence diversity and richness estimates [23]. Despite the differences between studies, many of the 8 predominant genera in infant saliva samples reported by Ling et al. [7], including *Streptococcus* (∼25%), *Prevotella* (∼20%), *Neisseria* (∼10%), *Haemophilus* (5–10%), *Porphyromonas* (5–10%), *Rothia* (∼5%), *Veillonella* (∼5%), and *Granulicatella* (∼5%), were also highly prevalent in our samples (62%, 0.72%, 4.9%, 2.6%, 0.7%, 3.2%, 15.3%, and 1.7% respectively). In the current study, an extremely high prevalence of *Streptococcus* and low prevalence of genera in the Bacteroidetes phyla [i.e., *Prevotella* (0.72%) and *Porphyromonas* (0.7%)] were observed. The prevalence of the other genera measured in our study were quite similar to that of previous studies. Recent studies have identified significant *Lactobacillus* [11], [12] and *Bifidobacterium* [11] populations in the saliva of children, but were ubiquitous but not highly prevalent in our population (0.04% and 0.02%, respectively).

Dominguez-Bello et al. [21] reported that microbiota populations in newborns were indistinguishable among sampling sites (skin, oral mucosa, and nasopharyngeal aspirate) <5 min of delivery, but were affected by mode of delivery. Those delivered by vaginal birth had bacterial populations having high similarity with the vaginal microbiota (dominated by *Lactobacillus*, *Prevotella*, and *Sneathia* spp.) of their mothers. In contrast, bacterial populations of infants delivered by Cesarean section more closely mimicked the skin-associated bacteria (dominated by *Staphylococcus*, *Corynebacterium*, and *Propionibacterium* spp.) of their mother. These bacterial populations are quite different than those reported in the current study and the other recent studies in young children [7]–[12].

Researchers have spent decades using traditional culture methods to study the microbial evolution of the mouth and the populations thought to contribute to oral diseases. Although significant progress has been made using such assays, recent technologies such as 454 pyrosequencing possess many advantages. Based on DNA content, these assays allow one to measure all microbial groups, distinguish specific strains from one another, and enable the study of the entire population structure rather than a few select taxa that can be cultured in the lab. Pyrosequencing technology has not only been used to study oral bacteria, but has been used to characterize other members of the oral microbial community, including fungi [24].

*Veillonella*, *Fusobacterium*, and *Actinomyces* have long been considered to be early colonizers of the oral cavity [25], [26], with others including *Streptococcus* establishing themselves after tooth eruption. Based on our data, the latter assumption appears to not be the case. Thus, the “window of infectivity” theory of acquiring dental pathogens, which was thought to occur between 19 and 33 months of age by Caufield et al. [27], should be reevaluated using the molecular techniques now available. Infant saliva in our experiment already contained most of the bacterial species necessary for oral biofilm development and disease (Table S2). For instance, many of the early colonizers in the *Streptococcus* (*S. sanguinis, S. mitis, S. gordonii*), *Veillonella* (*V. ratti, V. criceti, V. caviae, V. dispar*), *Actinomyces* (*A. odontolyticus, A. meyeri, A. graevenitzii*), and *Fusobacterium* (*F. periodonticum*) genera, taxa that are the first to coat the tooth surface and provide the foundation for plaque development [28], were relatively abundant in the saliva of our infant population. Late colonizers such as *Fusobacterium nucleatum, Eubacterium spp., Tannerella denticola, Porphyromonas gingivalis, Prevotella intermedia*, and *Selenomonas flueggei* were also present in infant saliva. While the prevalence of *S. mutans* was very low in the infant population (0.0001% of all species), their saliva contained other contributors to early childhood caries, including *Granulicatella elegans* (0.97%), *Veillonella parvula* (0.43%), *Streptococcus cristatus* (0.01%), *Bifidobacterium longum* (0.01%), and others [8]–[10]. Our data agree with that of Gizani [12] that the soft tissues of the oral cavity serve as reservoirs for oral pathogens, highlighting the importance of oral hygiene practices prior to tooth eruption.

The microbial evolution that occurs in the oral cavity between birth and tooth eruption, as teeth erupt, and as dietary changes occur (e.g., breastfeeding vs. formula feeding; from liquid to solid food; changes in nutrient profile), has not been well characterized. The great bacterial richness present in saliva of our edentulous infant population, and the fact that one infant exposed to solid foods clustered more closely to adults than other infants, demonstrate the need for a better understanding in this area. Because severe early childhood caries are related to early bacterial colonization, characterizing how dietary or other environmental factors alter the oral microbiota are greatly needed and may impact oral health strategies in the future.

## Materials and Methods

### Ethics statement

Written informed consent was obtained by all participants or their parents (for infants) in this study. All study procedures were approved by the University of Illinois Institutional Review Board (IRB) prior to participant recruitment.

### Subjects and sample collection

Five subject pairs (infant-primary care giver dyads) enrolled in the WIC Program at the Champaign-Urbana Public Health District, IL were recruited to participate in this experiment. The Special Supplemental Nutrition Program for WIC is a federal grant program provided from the United States Department of Agriculture Food and Nutrition Service. This program is intended for low income populations who are at a higher nutrition risk by offering supplemental nutrition, nutrition education, and screening and referrals to other social services. This population was selected because WIC children are at greater risk for many nutrition-related issues including oral disease. University of Illinois Institutional Review Board approved the study protocol prior to experimentation. The target population of infants was 2 to 6 mo of age and their primary care givers. After receiving written informed consent from parents at WIC office, 2–3 mL of saliva was collected from infants by research staff using sterile, disposable pipettes and placed into sterile cryogenic vials. Adults were asked to expectorate into sterile cryogenic vials with the average of 8 h of no food consumption for adults and 45 min for infants. All samples were placed into a refrigerator for 2–3 h and then transported to the laboratory and stored at −80°C until analyses.

### DNA extraction and PCR procedure

Salivary DNA was extracted using a slight modification of the method of Yu and Morrison [29] and the Qiagen Stool Mini Kit (Qiagen, Valencia, CA). The primary modification to the procedure was that instead of using a beadbeater, 0.006–0.01 g of 0.5 mm sterile glass beads (Sigma, St. Louis, MO) was added to 1 mL of saliva and vortexed to reduce DNA shearing. After extraction, DNA was cleaned and concentrated using a MinElute Reaction Cleanup Kit (Qiagen) and then quantified using an ND-1000 spectrophotometer (Nanodrop Technologies, Wilmington, DE). Amplification of the variable region 4–6 (V4–V6) of the bacterial 16S rDNA gene was then performed. Amplicon fusion primers were used to generate amplicons to allow for automated software identification of samples after pooling/multiplexing and pyrosequencing, a process also known as bar-coding. Fusion primers contained: 1) a directional GS FLX Titanium ‘Primer A’ sequence (CGTATCGCCTCCCTCGCGCCATCAG; forward primers) or ‘Primer B’ sequence (CTATGCGCCTTGCCAGCCCGCTCAG; reverse primers) at the 5-prime portion of the oligonucleotide; 2) a Multiplex Identifier (MID) that was unique to each sample; and 3) a Eubacterial-specific sequence for the V4–V6 region of the 16S rDNA gene, at the 3-prime end. We used the 16S universal Eubacterial primers 530F (5′-GTGCCAGCMGCNGCGG) and 1100R (5′-GGGTTNGNTCGTTG) because they have been shown to amplify a 600-bp region of the 16S rDNA gene [30]. The FastStart High Fidelity PCR System, dNTPack (Roche Applied Science, Indianapolis, IN) was used for PCR under the following conditions: 94°C for 3 min followed by 20 cycles of 94°C for 30 sec, 62°C for 45 sec, and 72°C for 1 min, and a final elongation step at 72°C for 2 min.

After PCR, the amplicons were purified using Agencourt® AMPure® XP according to manufacturer instructions (Beckman-Coulter, Inc., Brea, CA). Prior to pyrosequencing, DNA quality was assessed using a 2100 Bioanalyzer (Agilent, Santa Clara, CA). Sequencing of the PCR products was performed at the W. M. Keck Center for Biotechnology at the University of Illinois using a 454 Genome Sequencer using FLX titanium reagents (Roche Applied Science). After sequencing was completed, all reads were scored for quality and any poor quality reads and primer dimers were removed.

### Data analysis

#### Diversity estimates

To estimate total bacterial diversity of DNA samples in a comparable manner, sequences were selected which read from the 1100R direction. Sequences were depleted of barcodes primers, chimeras, plastid, mitochondrial, and any non-16S bacterial reads. Chimeras were depleted using Black Box Chimera Check (B2C2) as described previously [31]. Sequences <350 bp were also depleted. Because sequence number may impact diversity estimates, an equal number of high quality sequences were used for each sample. A total of4,750 sequences from each sample were selected based upon highest average quality score, sequences trimmed to 350 bp and aligned with MUSCLE [32]. A distance matrix was calculated from the alignment with PHYLIP [33]. Operational Taxonomical Units (OTU) then were assigned by MOTHUR using the read.otu command [16], [34]. Using MOTHUR output (rarefaction.single command), the maximum observed rarefaction OTU values were generated and rarefaction curves were also modeled with the Richard's equation as described previously [35], [36] to generate maximum predicted OTU. Rarefaction values were also generated using QIIME using suggested workflow including denoising. PD-whole tree outputs at 3% divergence were utilized to generate rarefaction curves and as above were modeled using the Richard's equation to generate maximum predicted OTU for each of the samples.

To evaluate bacterial ID community structure, Phred20 quality reads, including both 530F and 1100R oriented (each analyzed separately), were trimmed to remove tags and primers sequence collections, were then depleted of chimera, plastid, mitochondrial, and any non-16S reads (<70% identity to any known high quality 16S sequence) and sequences <250 bp were also depleted. The final sequences 425,239 (mean = 42,523) for infants and 330,560 (mean = 41,320) for adults were evaluated using Kraken (www.krakenblast.com) against a 10-21-10 version database curated from NCBI to include >300,000 high quality 16S bacteria and archaeal sequences as well as quality control screening sequences for mitochondria, plastid, and chloroplast screening sequences. Blast output based upon top hit designations were compiled to generate percentage files at each taxonomic level as described previously [37]–[39]. Community structure based upon taxonomic analyses were utilized to generate dual hierarchal dendrograms. Dendrograms for phyla show all phyla, while dendrograms for genera illustrate the top 50 genera based upon highest average percentage among both adults and infants. ANOVA with Bonferroni post-hoc analyses were utilized to define significance between adults and infants for each phyla and the top 50 genera (highest abundance and most ubiquitous).

UniFrac was also used to evaluate the relatedness of samples within the same age as described previously using the 4750 average high quality sequences described above as well as with QIIME. No discrepancy was observed between the two methods so the prior method (high quality sequence method) was utilized to compare samples using principal component analysis. In short, the high quality 1100R sequences prepared as described above were aligned using MUSCLE, and optimized tree was generated. This tree served as the input tree for UniFrac. Weighed and normalized Principal Component Analysis (PCA) was performed to evaluate similarity among samples, where each sample represents an environment. Based upon primary 3 vectors significance was evaluated using two tailed T-test.

## Supporting Information

Table S1 All bacterial genera present in baby and mother/primary care giver saliva samples.
(DOCX)

Table S2 All bacterial species present in baby and mother/primary care giver saliva samples.
(DOCX)
