# Supplementary material for: Comparative Analysis of Salivary Bacterial Microbiome Diversity in Edentulous Infants and Their Mothers or Primary Care Givers Using Pyrosequencing
Source: PLoS One. 2011 Aug 10;6(8):e23503. doi: 10.1371/journal.pone.0023503 (PMC3154475; doi:10.1371/journal.pone.0023503)
Supplement: Table S1 — All bacterial genera present in baby and mother/primary care giver saliva samples. (DOCX) [file pone.0023503.s001.docx]

| **Table S1. All bacterial genera present in baby and mother/primary care giver saliva samples** | | |
| --- | --- | --- |
|  |  |  |
| **Genus Name** | **Baby (% of sequences)** | **Mother/Care Giver (% of sequences)** |
| Streptococcus | 62.21298 | 20.39327 |
| Haemophilus | 2.62817 | 16.51318 |
| Neisseria | 4.93050 | 11.82864 |
| Veillonella | 15.35579 | 11.59061 |
| Fusobacterium | 1.24721 | 6.48661 |
| Oribacterium | 0.37979 | 6.30275 |
| Rothia | 3.28115 | 3.67679 |
| Treponema | 0.00019 | 2.76438 |
| Actinomyces | 0.44159 | 2.75947 |
| Campylobacter | 0.24866 | 2.43238 |
| Granulicatella | 1.71557 | 1.56470 |
| Eubacterium | 0.20729 | 1.34001 |
| Gemella | 2.03445 | 1.25836 |
| Leptotrichia | 1.40682 | 0.88623 |
| Megasphaera | 0.09816 | 0.80385 |
| Filifactor | 0.00353 | 0.77658 |
| Selenomonas | 0.00496 | 0.77285 |
| Atopobium | 0.08706 | 0.60666 |
| Aggregatibacter | 0.00354 | 0.55168 |
| Prevotella | 0.72047 | 0.45348 |
| Catonella | 0.01891 | 0.42981 |
| Lautropia | 0.00018 | 0.41063 |
| Dialister | 0.00342 | 0.38969 |
| Parvimonas | 0.00000 | 0.37719 |
| Mycoplasma | 0.00000 | 0.35537 |
| Solobacterium | 0.12625 | 0.34960 |
| Clostridiales (genus) | 0.01606 | 0.31885 |
| Lachnospiraceae (genus) | 0.01433 | 0.30895 |
| Scardovia | 0.00000 | 0.24537 |
| Peptostreptococcus | 0.00615 | 0.23976 |
| Mogibacterium | 0.01079 | 0.20634 |
| Kingella | 0.01658 | 0.20149 |
| Mycoplasmataceae (genus) | 0.00000 | 0.18388 |
| Veillonellaceae (genus) | 0.00055 | 0.18061 |
| Bulleidia | 0.00000 | 0.16424 |
| Aminobacterium | 0.00000 | 0.16395 |
| Corynebacterium | 0.00878 | 0.14061 |
| Bergeyella | 0.76258 | 0.10911 |
| Actinobacillus | 0.03032 | 0.10734 |
| Lactobacillus | 0.01622 | 0.07915 |
| Eubacteriaceae (genus) | 0.00000 | 0.07528 |
| Peptostreptococcaceae (genus) | 0.00000 | 0.07407 |
| Lactococcus | 0.02880 | 0.05899 |
| Eikenella | 0.00175 | 0.05316 |
| Porphyromonas | 0.69314 | 0.05240 |
| Pyramidobacter | 0.00000 | 0.05013 |
| Clostridium | 0.08140 | 0.04927 |
| Shuttleworthia | 0.00000 | 0.04184 |
| Synergistes | 0.00000 | 0.04137 |
| Simonsiella | 0.01978 | 0.03513 |
| Actinobaculum | 0.00000 | 0.03464 |
| Peptococcus | 0.00000 | 0.03304 |
| Bacillus | 0.11811 | 0.02935 |
| Bifidobacterium | 0.01375 | 0.02788 |
| Pasteurellaceae (genus) | 0.00416 | 0.02651 |
| Staphylococcus | 0.03456 | 0.02581 |
| Anaeroglobus | 0.00000 | 0.02546 |
| Bifidobacteriaceae | 0.00000 | 0.02099 |
| Pseudoramibacter | 0.00052 | 0.01975 |
| Arthrobacter | 0.01522 | 0.01933 |
| Asteroleplasma | 0.00000 | 0.01814 |
| Anaerobaculum | 0.00000 | 0.01500 |
| Clostridia (genus) | 0.00000 | 0.01402 |
| Micrococcaceae (genus) | 0.07376 | 0.01248 |
| Propionibacterium | 0.01985 | 0.01244 |
| Cardiobacterium | 0.00000 | 0.01233 |
| Capnocytophaga | 0.00116 | 0.01002 |
| Moraxellaceae (genus) | 0.10594 | 0.00943 |
| Enterococcus | 0.02237 | 0.00940 |
| Roseburia | 0.00020 | 0.00915 |
| Carnobacterium | 0.00504 | 0.00874 |
| Pilibacter | 0.05063 | 0.00844 |
| Thermovirga | 0.00000 | 0.00822 |
| Johnsonella | 0.00000 | 0.00818 |
| Lacticigenium | 0.00523 | 0.00785 |
| Bavariicoccus | 0.00893 | 0.00744 |
| Alysiella | 0.00937 | 0.00720 |
| Oxalobacteraceae (genus) | 0.00020 | 0.00697 |
| Vagococcus | 0.01095 | 0.00691 |
| Pasteurella | 0.00315 | 0.00669 |
| Centipeda | 0.00000 | 0.00645 |
| Trichococcus | 0.00210 | 0.00634 |
| Ruminococcus | 0.00342 | 0.00618 |
| Syntrophaceticus | 0.00000 | 0.00606 |
| Serratia | 0.00097 | 0.00556 |
| Desulfomicrobium | 0.00000 | 0.00543 |
| Cryptobacterium | 0.00000 | 0.00536 |
| Hallella | 0.00000 | 0.00522 |
| Dethiosulfatibacter | 0.00000 | 0.00516 |
| Paenibacillus | 0.02669 | 0.00512 |
| Anaerorhabdus | 0.00000 | 0.00478 |
| Desulfobulbus | 0.00000 | 0.00475 |
| Mannheimia | 0.00000 | 0.00423 |
| Kocuria | 0.00463 | 0.00409 |
| Tannerella | 0.00000 | 0.00397 |
| Olsenella | 0.00000 | 0.00394 |
| Candidatus Wolinella | 0.00000 | 0.00380 |
| Bisgaardia | 0.00063 | 0.00379 |
| Dermacoccus | 0.00952 | 0.00377 |
| Streptobacillus | 0.09414 | 0.00367 |
| Anaplasma | 0.00000 | 0.00363 |
| Schwartzia | 0.00044 | 0.00362 |
| Mitsuokella | 0.00024 | 0.00357 |
| Clostridiaceae (genus) | 0.00057 | 0.00340 |
| Gardnerella | 0.00000 | 0.00336 |
| Lactobacillus (Erysipelotrichaceae) | 0.00000 | 0.00331 |
| Phascolarctobacterium | 0.00945 | 0.00330 |
| Serinicoccus | 0.00639 | 0.00327 |
| Bacteroidaceae (genus) | 0.00000 | 0.00323 |
| Micrococcus | 0.01024 | 0.00314 |
| Parascardovia | 0.00000 | 0.00312 |
| Butyrivibrio | 0.00037 | 0.00294 |
| Vibrio | 0.00020 | 0.00291 |
| Imtechium | 0.00487 | 0.00279 |
| Nesterenkonia | 0.00604 | 0.00273 |
| Abiotrophia | 0.00246 | 0.00271 |
| Cohnella | 0.00000 | 0.00264 |
| Enterobacter | 0.02497 | 0.00263 |
| Bacteroides | 0.00113 | 0.00256 |
| Isobaculum | 0.00064 | 0.00253 |
| Brevibacillus | 0.00167 | 0.00240 |
| Cloacibacterium | 0.00615 | 0.00226 |
| Peptoniphilus | 0.00000 | 0.00225 |
| Chitinilyticum | 0.00348 | 0.00220 |
| Acidovorax | 0.00805 | 0.00220 |
| Dietzia | 0.00371 | 0.00208 |
| Hahella | 0.00000 | 0.00207 |
| Alloscardovia | 0.00000 | 0.00202 |
| Blautia | 0.00108 | 0.00191 |
| Conchiformibius | 0.00039 | 0.00190 |
| Viridibacillus | 0.01777 | 0.00189 |
| Sphaerochaeta | 0.00000 | 0.00181 |
| Aneurinibacillus | 0.00803 | 0.00176 |
| Chryseobacterium | 0.01425 | 0.00173 |
| Tetrasphaera | 0.00121 | 0.00172 |
| Bacteroidales (genus) | 0.00000 | 0.00169 |
| Shewanella | 0.00078 | 0.00164 |
| Kineosporiaceae (genus) | 0.00052 | 0.00163 |
| Alkalimonas | 0.00045 | 0.00163 |
| Anaerovorax | 0.00000 | 0.00152 |
| Trichlorobacter | 0.00073 | 0.00150 |
| Chitinibacter | 0.00116 | 0.00148 |
| Aerococcus | 0.00114 | 0.00148 |
| Phocoenobacter | 0.00098 | 0.00144 |
| Dermabacter | 0.00079 | 0.00141 |
| Moryella | 0.00019 | 0.00140 |
| Yaniella | 0.00117 | 0.00139 |
| Arcanobacterium | 0.00059 | 0.00135 |
| Pigmentiphaga | 0.00000 | 0.00132 |
| Sporosarcina | 0.00026 | 0.00130 |
| Dethiosulfovibrio | 0.00000 | 0.00130 |
| Acidithiobacillus | 0.00037 | 0.00126 |
| Acetonema | 0.01322 | 0.00126 |
| Ammoniphilus | 0.00050 | 0.00126 |
| Sphingomonas | 0.00111 | 0.00125 |
| Tissierella | 0.00026 | 0.00122 |
| Bergeriella | 0.00018 | 0.00115 |
| Kineosphaera | 0.00024 | 0.00113 |
| Syntrophomonas | 0.00000 | 0.00110 |
| Nicoletella | 0.00000 | 0.00106 |
| Acholeplasma | 0.00000 | 0.00105 |
| Spirochaeta | 0.00000 | 0.00103 |
| Nitrincola | 0.00039 | 0.00102 |
| Pseudomonas | 0.00344 | 0.00102 |
| Enterobacteriaceae (genus) | 0.00067 | 0.00101 |
| Prevotellaceae (genus) | 0.00000 | 0.00101 |
| Vibrionaceae (genus) | 0.00000 | 0.00100 |
| Haloplasma | 0.00000 | 0.00088 |
| Kurthia | 0.00570 | 0.00086 |
| Propionispora | 0.00044 | 0.00086 |
| Anaerofustis | 0.00000 | 0.00086 |
| Acinetobacter | 0.19934 | 0.00084 |
| Neptunomonas | 0.00059 | 0.00082 |
| Allobaculum | 0.00134 | 0.00080 |
| Heliobacteriaceae (genus) | 0.00000 | 0.00080 |
| Diaphorobacter | 0.00174 | 0.00080 |
| Anaerosinus | 0.00018 | 0.00079 |
| Sneathia | 0.00699 | 0.00066 |
| Acetobacterium | 0.00020 | 0.00066 |
| Desulfovibrio | 0.00000 | 0.00066 |
| Atopobacter | 0.00563 | 0.00064 |
| Psychrosinus | 0.00019 | 0.00064 |
| Avibacterium | 0.00000 | 0.00064 |
| Turicibacter | 0.00000 | 0.00064 |
| Sedimentibacter | 0.00020 | 0.00062 |
| Pontibacter | 0.00000 | 0.00062 |
| Bibersteinia | 0.00000 | 0.00060 |
| Rhizobiales (genus) | 0.00000 | 0.00060 |
| Citrobacter | 0.00038 | 0.00060 |
| Methylibium | 0.00000 | 0.00060 |
| Gulbenkiania | 0.00000 | 0.00060 |
| Methylobacterium | 0.00063 | 0.00060 |
| Anaerococcus | 0.00059 | 0.00060 |
| Propionivibrio | 0.00000 | 0.00060 |
| Salinivibrio | 0.00000 | 0.00060 |
| Carnobacteriaceae (genus) | 0.00024 | 0.00060 |
| Pelosinus | 0.00069 | 0.00060 |
| Marinobacterium | 0.00020 | 0.00060 |
| Jonquetella | 0.00000 | 0.00060 |
| Lactobacillales (genus) | 0.00820 | 0.00057 |
| Lentzea | 0.00053 | 0.00057 |
| Dermatophilaceae (genus) | 0.00017 | 0.00057 |
| Demetria | 0.00000 | 0.00057 |
| Catabacter | 0.00065 | 0.00044 |
| Beggiatoa | 0.00057 | 0.00044 |
| Actinomycetospora | 0.00000 | 0.00044 |
| Actinopolyspora | 0.00000 | 0.00044 |
| Alishewanella | 0.00000 | 0.00044 |
| Helicobacter | 0.00000 | 0.00044 |
| Methylocystaceae (genus) | 0.00000 | 0.00044 |
| Shimazuella | 0.00000 | 0.00044 |
| Sporomusa | 0.00000 | 0.00044 |
| Tindallia | 0.00000 | 0.00044 |
| Peptococcaceae (genus) | 0.00378 | 0.00042 |
| Rhizobium | 0.00025 | 0.00042 |
| Burkholderiales (genus) | 0.00020 | 0.00042 |
| Collimonas | 0.00000 | 0.00042 |
| Lactonifactor | 0.00000 | 0.00042 |
| Mytilidae | 0.00232 | 0.00042 |
| Gigantidas | 0.00082 | 0.00042 |
| Tetragenococcus | 0.00057 | 0.00042 |
| Ornithinicoccus | 0.00155 | 0.00040 |
| Flavobacteriaceae (genus) | 0.00101 | 0.00040 |
| Acetobacter | 0.00000 | 0.00040 |
| Herbaspirillum | 0.00000 | 0.00040 |
| Hyphomonas | 0.00000 | 0.00040 |
| Coprococcus | 0.00019 | 0.00040 |
| Thermoanaerobacter | 0.00000 | 0.00040 |
| Pseudobutyrivibrio | 0.00000 | 0.00040 |
| Proteiniborus | 0.00000 | 0.00040 |
| Photobacterium | 0.00000 | 0.00039 |
| Streptococcaceae (genus) | 0.00000 | 0.00039 |
| Bacillales (genus) | 0.00019 | 0.00039 |
| Lonepinella | 0.00000 | 0.00039 |
| Bacillaceae (genus) | 0.00168 | 0.00022 |
| Comamonas | 0.00057 | 0.00022 |
| Thalassomonas | 0.00052 | 0.00022 |
| Vitreoscilla | 0.00045 | 0.00022 |
| Desulfonispora | 0.00044 | 0.00022 |
| Geobacter | 0.00037 | 0.00022 |
| Marihabitans | 0.00037 | 0.00022 |
| Desulfosporosinus | 0.00026 | 0.00022 |
| Moorella | 0.00026 | 0.00022 |
| Gracilibacillus | 0.00020 | 0.00022 |
| Ectothiorhodospira | 0.00018 | 0.00022 |
| Achromobacter | 0.00000 | 0.00022 |
| Alcaligenes | 0.00000 | 0.00022 |
| Aminiphilus | 0.00000 | 0.00022 |
| Aquaspirillum | 0.00000 | 0.00022 |
| Candidatus Prevotella | 0.00000 | 0.00022 |
| Cytophaga | 0.00000 | 0.00022 |
| Desulfonatronovibrio | 0.00000 | 0.00022 |
| Fusibacter | 0.00000 | 0.00022 |
| Geobacteraceae (genus) | 0.00000 | 0.00022 |
| Haliea | 0.00000 | 0.00022 |
| Hyphomicrobiaceae (genus) | 0.00000 | 0.00022 |
| Idiomarina | 0.00000 | 0.00022 |
| Lachnospira | 0.00000 | 0.00022 |
| Leuconostoc | 0.00000 | 0.00022 |
| Lutispora | 0.00000 | 0.00022 |
| Lysobacter | 0.00000 | 0.00022 |
| Marinibacillus | 0.00000 | 0.00022 |
| Marinilactibacillus | 0.00000 | 0.00022 |
| Neisseriaceae (genus) | 0.00000 | 0.00022 |
| Oceanospirillum | 0.00000 | 0.00022 |
| Pullulanibacillus | 0.00000 | 0.00022 |
| Rhodanobacter | 0.00000 | 0.00022 |
| Rhodoferax | 0.00000 | 0.00022 |
| Sinomonas | 0.00000 | 0.00022 |
| Tepidimicrobium | 0.00000 | 0.00022 |
| Thalassolituus | 0.00000 | 0.00022 |
| Wolinella | 0.00000 | 0.00022 |
| Zymobacter | 0.00000 | 0.00022 |
| Desemzia | 0.00237 | 0.00020 |
| Intrasporangium | 0.00068 | 0.00020 |
| Anaerosporobacter | 0.00041 | 0.00020 |
| Bathymodiolus | 0.00019 | 0.00020 |
| Gallibacterium | 0.00019 | 0.00020 |
| Serinibacter | 0.00017 | 0.00020 |
| Alkaliphilus | 0.00000 | 0.00020 |
| Blastomonas | 0.00000 | 0.00020 |
| Devosia | 0.00000 | 0.00020 |
| Gracilibacter | 0.00000 | 0.00020 |
| Kribbia | 0.00000 | 0.00020 |
| Ochrobactrum | 0.00000 | 0.00020 |
| Olivibacter | 0.00000 | 0.00020 |
| Quadrisphaera | 0.00000 | 0.00020 |
| Ruegeria | 0.00000 | 0.00020 |
| Sphingobium | 0.00000 | 0.00020 |
| Halomonas | 0.00039 | 0.00020 |
| Anaerobiospirillum | 0.00000 | 0.00020 |
| Anaeromusa | 0.00000 | 0.00020 |
| Caryophanon | 0.00000 | 0.00020 |
| Chitinophagaceae (genus) | 0.00000 | 0.00020 |
| Coraliomargarita | 0.00000 | 0.00020 |
| Dendrosporobacter | 0.00000 | 0.00020 |
| Eubacterium (Erysipelotrichaceae) | 0.00000 | 0.00020 |
| Luteimonas | 0.00000 | 0.00020 |
| Natronincola | 0.00000 | 0.00020 |
| Paraferrimonas | 0.00000 | 0.00020 |
| Paucimonas | 0.00000 | 0.00020 |
| Thiobacillus | 0.00000 | 0.00020 |
| Aeromonas | 0.00711 | 0.00000 |
| Klebsiella | 0.00583 | 0.00000 |
| Pantoea | 0.00477 | 0.00000 |
| Actinomycetales (genus) | 0.00398 | 0.00000 |
| Geobacillus | 0.00320 | 0.00000 |
| Riemerella | 0.00276 | 0.00000 |
| Thermus | 0.00261 | 0.00000 |
| Xanthomonas | 0.00190 | 0.00000 |
| Acetivibrio | 0.00168 | 0.00000 |
| Agrobacterium | 0.00153 | 0.00000 |
| Haloanella | 0.00138 | 0.00000 |
| Plesiomonas | 0.00130 | 0.00000 |
| Bradyrhizobium | 0.00114 | 0.00000 |
| Escherichia | 0.00113 | 0.00000 |
| Brevibacterium | 0.00109 | 0.00000 |
| Cronobacter | 0.00104 | 0.00000 |
| Sporolactobacillus | 0.00100 | 0.00000 |
| Erwinia | 0.00097 | 0.00000 |
| Microbacterium | 0.00093 | 0.00000 |
| Pseudoxanthomonas | 0.00092 | 0.00000 |
| Aquabacterium | 0.00088 | 0.00000 |
| Flavobacterium | 0.00082 | 0.00000 |
| Desulfitobacterium | 0.00081 | 0.00000 |
| Paracoccus | 0.00080 | 0.00000 |
| Brevundimonas | 0.00078 | 0.00000 |
| Micropruina | 0.00077 | 0.00000 |
| Salinibacillus | 0.00077 | 0.00000 |
| Anaerobacillus | 0.00071 | 0.00000 |
| Dermatophilus | 0.00065 | 0.00000 |
| Deinococcus | 0.00061 | 0.00000 |
| Fusobacteriaceae (genus) | 0.00061 | 0.00000 |
| Stenotrophomonas | 0.00059 | 0.00000 |
| Weissella | 0.00057 | 0.00000 |
| Niastella | 0.00057 | 0.00000 |
| Anoxybacillus | 0.00052 | 0.00000 |
| Sebaldella | 0.00052 | 0.00000 |
| Pseudonocardiaceae (genus) | 0.00042 | 0.00000 |
| Pectobacterium | 0.00039 | 0.00000 |
| Psychrobacter | 0.00039 | 0.00000 |
| Salmonella | 0.00038 | 0.00000 |
| Sediminibacterium | 0.00038 | 0.00000 |
| Flavisolibacter | 0.00037 | 0.00000 |
| Morganella | 0.00037 | 0.00000 |
| Cellulomonas | 0.00037 | 0.00000 |
| Rubrobacter | 0.00037 | 0.00000 |
| Edwardsiella | 0.00037 | 0.00000 |
| Oxalophagus | 0.00037 | 0.00000 |
| Ornithinimicrobium | 0.00036 | 0.00000 |
| Acaricomes | 0.00036 | 0.00000 |
| Desulfocaldus | 0.00036 | 0.00000 |
| Oceanobacillus | 0.00036 | 0.00000 |
| Sediminibacillus | 0.00036 | 0.00000 |
| Dermacoccaceae (genus) | 0.00034 | 0.00000 |
| Phycicoccus | 0.00034 | 0.00000 |
| Rummeliibacillus | 0.00034 | 0.00000 |
| Amphibacillus | 0.00026 | 0.00000 |
| Cedecea | 0.00026 | 0.00000 |
| Sanguibacter | 0.00026 | 0.00000 |
| Caulobacter | 0.00025 | 0.00000 |
| Oscillospiraceae (genus) | 0.00025 | 0.00000 |
| Brochothrix | 0.00024 | 0.00000 |
| Alkanindiges | 0.00020 | 0.00000 |
| Aquamonas | 0.00020 | 0.00000 |
| Aquisalimonas | 0.00020 | 0.00000 |
| Citreimonas | 0.00020 | 0.00000 |
| Dorea | 0.00020 | 0.00000 |
| Leminorella | 0.00020 | 0.00000 |
| Marinospirillum | 0.00020 | 0.00000 |
| Mitsuaria | 0.00020 | 0.00000 |
| Novosphingobium | 0.00020 | 0.00000 |
| Planomicrobium | 0.00020 | 0.00000 |
| Saccharococcus | 0.00020 | 0.00000 |
| Sphingobacteriales (genus) | 0.00020 | 0.00000 |
| Sphingobacterium | 0.00020 | 0.00000 |
| Trabulsiella | 0.00020 | 0.00000 |
| Brachybacterium | 0.00019 | 0.00000 |
| Chelatococcus | 0.00019 | 0.00000 |
| Epulopiscium | 0.00019 | 0.00000 |
| Gemmatimonas | 0.00019 | 0.00000 |
| Geodermatophilaceae (genus) | 0.00019 | 0.00000 |
| Kluyvera | 0.00019 | 0.00000 |
| Roseomonas | 0.00019 | 0.00000 |
| Weeksella | 0.00019 | 0.00000 |
| Bogoriella | 0.00018 | 0.00000 |
| Halobacillus | 0.00018 | 0.00000 |
| Hydrogenophaga | 0.00018 | 0.00000 |
| Janibacter | 0.00018 | 0.00000 |
| Promicromonospora | 0.00018 | 0.00000 |
| Rhodopseudomonas | 0.00018 | 0.00000 |
| Rhodospirillales (genus) | 0.00018 | 0.00000 |
| Shigella | 0.00018 | 0.00000 |
| Siphonobacter | 0.00018 | 0.00000 |
| Actinotalea | 0.00017 | 0.00000 |
| Salirhabdus | 0.00017 | 0.00000 |
| Solibacillus | 0.00017 | 0.00000 |
