# Supplementary material for: Comparative Analysis of Salivary Bacterial Microbiome Diversity in Edentulous Infants and Their Mothers or Primary Care Givers Using Pyrosequencing
Source: PLoS One. 2011 Aug 10;6(8):e23503. doi: 10.1371/journal.pone.0023503 (PMC3154475; doi:10.1371/journal.pone.0023503)
Supplement: Table S2 — All bacterial species present in baby and mother/primary care giver saliva samples. (DOCX) [file pone.0023503.s002.docx]

| **Table S2. All bacterial species present in baby and mother/primary care giver saliva samples** | | |
| --- | --- | --- |
|  |  |  |
| **Species Name** | **Baby (% of sequences)** | **Mother/Care Giver (% of sequences)** |
| Streptococcus mitis | 50.5597 | 9.57340 |
| Veillonella ratti | 7.5263 | 0.06774 |
| Neisseria animalis | 4.5012 | 8.31761 |
| Veillonella criceti | 3.0949 | 0.03690 |
| Streptococcus parasanguinis | 2.8145 | 5.11292 |
| Veillonella caviae | 2.7217 | 0.90077 |
| TM7 genera incertae sedis uncultured | 2.6930 | 0.79060 |
| Veillonella dispar | 2.4870 | 10.17773 |
| Gemella morbillorum | 1.9114 | 1.20910 |
| Streptococcus pneumoniae | 1.9087 | 0.37994 |
| Streptococcus peroris | 1.7386 | 0.48333 |
| Haemophilus parainfluenzae | 1.4475 | 13.32992 |
| Rothia amarae | 1.3530 | 1.58268 |
| Rothia mucilaginosa | 1.1322 | 1.43130 |
| Fusobacterium nucleatum | 1.0578 | 5.77445 |
| Granulicatella elegans | 0.9673 | 0.12812 |
| Leptotrichia goodfellowii | 0.8218 | 0.05599 |
| Streptococcus thermophilus | 0.6900 | 2.07495 |
| Porphyromonas catoniae | 0.6597 | 0.01817 |
| Haemophilus influenzae | 0.6235 | 0.82226 |
| Chryseobacterium joostei | 0.5133 | 0.01114 |
| Veillonella parvula | 0.4278 | 1.18813 |
| Granulicatella adiacens | 0.3795 | 0.99252 |
| Veillonella montpellierensis | 0.3759 | 0.00549 |
| Leptotrichia shahii | 0.3748 | 0.29093 |
| Oribacterium sinus | 0.3705 | 4.45011 |
| Bisgaard Taxon | 0.3612 | 2.07477 |
| Actinomyces odontolyticus | 0.3009 | 2.06092 |
| Bacteroides intestinalis | 0.3008 | 0.00443 |
| Campylobacter concisus | 0.2390 | 1.42756 |
| Prevotella veroralis | 0.2315 | 0.20675 |
| Granulicatella balaenopterae | 0.2237 | 0.32166 |
| Veillonella atypica | 0.2138 | 0.71089 |
| Streptococcus sanguinis | 0.1977 | 0.59370 |
| Streptococcus pseudopneumoniae | 0.1954 | 0.07442 |
| Rothia dentocariosa | 0.1894 | 0.44489 |
| Rothia aeria | 0.1804 | 0.17727 |
| Rothia nasimurium | 0.1652 | 0.06343 |
| Viridibacillus arvi | 0.1637 | 0.02314 |
| Streptococcus infantis | 0.1591 | 0.00887 |
| Enterococcus italicus | 0.1536 | 0.10898 |
| Bulleidia extructa | 0.1259 | 0.49283 |
| Haemophilus pittmaniae | 0.1112 | 0.36592 |
| Streptococcus sinensis | 0.1092 | 0.16182 |
| Megasphaera micronuciformis | 0.1072 | 0.86410 |
| Leptotrichia trevisanii | 0.1061 | 0.26483 |
| Chryseobacterium shigense | 0.1006 | 0.00480 |
| Moraxella canis | 0.0929 | 0.00000 |
| Streptobacillus moniliformis | 0.0911 | 0.04000 |
| Atopobium parvulum | 0.0838 | 0.50336 |
| Lachnospiraceae oral | 0.0838 | 2.17702 |
| Clostridium thermocellum | 0.0807 | 0.02262 |
| Leptotrichia hofstadii | 0.0797 | 0.11601 |
| Serinicoccus marinus | 0.0784 | 0.01997 |
| Eubacterium saburreum-like | 0.0770 | 0.27719 |
| Fusobacterium periodonticum | 0.0727 | 0.07983 |
| Acinetobacter johnsonii | 0.0725 | 0.00016 |
| Enterococcus termitis | 0.0639 | 0.00686 |
| Riemerella anatipestifer | 0.0590 | 0.06560 |
| Pilibacter termitis | 0.0563 | 0.00930 |
| Actinomyces meyeri | 0.0554 | 0.25288 |
| Actinomyces graevenitzii | 0.0508 | 0.16193 |
| Acinetobacter schindleri | 0.0489 | 0.00018 |
| Chryseobacterium hispanicum | 0.0488 | 0.01573 |
| Neisseria lactamica | 0.0463 | 1.39019 |
| Bacillus subtilis | 0.0454 | 0.00677 |
| Neisseria weaveri | 0.0440 | 0.08023 |
| Neisseria flavescens | 0.0435 | 0.07087 |
| Pasteurella aerogenes | 0.0428 | 0.14836 |
| Prevotella bivia | 0.0420 | 0.00016 |
| Prevotella melaninogenica | 0.0384 | 0.02101 |
| Clostridium citroniae | 0.0371 | 0.00980 |
| Streptococcus phocae | 0.0364 | 0.01567 |
| Acinetobacter lwoffii | 0.0342 | 0.00000 |
| Chryseobacterium soldanellicola | 0.0314 | 0.00827 |
| Streptococcus gordonii | 0.0312 | 0.20275 |
| Kocuria palustris | 0.0291 | 0.02093 |
| Chryseobacterium vrystaatense | 0.0277 | 0.00000 |
| Neisseria canis | 0.0236 | 0.04879 |
| Lactococcus lactis | 0.0231 | 0.07574 |
| Propionibacterium acnes | 0.0228 | 0.00299 |
| Nesterenkonia lacusekhoensis | 0.0226 | 0.00768 |
| Actinobacillus porcinus | 0.0219 | 0.11718 |
| Serratia marcescens | 0.0213 | 0.10115 |
| Fusobacterium canifelinum | 0.0213 | 0.10186 |
| Simonsiella muelleri | 0.0199 | 0.03951 |
| Streptococcus oligofermentans | 0.0199 | 0.07928 |
| Catonella morbi | 0.0195 | 0.46318 |
| Actinomyces cardiffensis | 0.0187 | 0.05491 |
| Elizabethkingia meningoseptica | 0.0183 | 0.00669 |
| Enterococcus mundtii | 0.0182 | 0.00147 |
| Filifactor alocis | 0.0162 | 0.02781 |
| Fusobacterium equinum | 0.0158 | 0.00330 |
| Gemella palaticanis | 0.0158 | 0.00976 |
| Actinobacillus pleuropneumoniae | 0.0156 | 0.37941 |
| Streptococcus agalactiae | 0.0153 | 0.00952 |
| Streptococcus cristatus | 0.0143 | 0.11763 |
| Phascolarctobacterium faecium | 0.0134 | 0.00431 |
| Kingella denitrificans | 0.0132 | 0.04995 |
| Gemella sanguinis | 0.0129 | 0.02072 |
| Enterobacter cloacae | 0.0127 | 0.00540 |
| Acinetobacter baumannii | 0.0124 | 0.00000 |
| Prevotella oulorum | 0.0123 | 0.00331 |
| Clostridium cellobioparum | 0.0123 | 0.06343 |
| Vagococcus lutrae | 0.0121 | 0.00829 |
| Kingella kingae | 0.0119 | 0.02551 |
| Clostridium hathewayi | 0.0118 | 0.00740 |
| Staphylococcus capitis | 0.0116 | 0.00033 |
| Tetragenococcus koreensis | 0.0116 | 0.00053 |
| Staphylococcus hominis | 0.0113 | 0.00000 |
| Arcanobacterium pluranimalium | 0.0113 | 0.00532 |
| Streptococcus suis | 0.0113 | 0.04248 |
| Fusobacterium russii | 0.0112 | 0.00293 |
| Dialister propionicifaciens | 0.0111 | 0.00955 |
| Campylobacter rectus | 0.0110 | 0.43833 |
| Actinotalea fermentans | 0.0107 | 0.00099 |
| Actinomyces howellii | 0.0104 | 0.01736 |
| Mogibacterium neglectum | 0.0104 | 0.11045 |
| Alysiella filiformis | 0.0101 | 0.01914 |
| Kluyvera intermedia | 0.0098 | 0.00000 |
| Prevotella salivae | 0.0096 | 0.00635 |
| Enterococcus devriesei | 0.0093 | 0.01208 |
| Moraxella oblonga | 0.0088 | 0.00053 |
| Prevotella oralis | 0.0088 | 0.02878 |
| Enhydrobacter aerosaccus | 0.0087 | 0.00930 |
| Streptococcus marimammalium | 0.0085 | 0.00909 |
| Aneurinibacillus thermoaerophilus | 0.0083 | 0.00053 |
| Chryseobacterium taichungense | 0.0083 | 0.00053 |
| Prevotella shahii | 0.0080 | 0.00084 |
| Bifidobacterium longum | 0.0079 | 0.00683 |
| Lactobacillus delbrueckii | 0.0076 | 0.00161 |
| Vagococcus carniphilus | 0.0074 | 0.00195 |
| Peptostreptococcus stomatis | 0.0071 | 0.18773 |
| Paenibacillus rhizosphaerae | 0.0071 | 0.00018 |
| Riemerella columbina | 0.0065 | 0.00244 |
| Streptococcus dysgalactiae | 0.0062 | 0.00202 |
| Clostridium josui | 0.0061 | 0.00383 |
| Bacteroides thetaiotaomicron | 0.0061 | 0.00063 |
| Streptococcus infantarius | 0.0059 | 0.00689 |
| Isobaculum melis | 0.0058 | 0.00740 |
| Acidovorax temperans | 0.0058 | 0.00218 |
| Bacillus koreensis | 0.0058 | 0.00018 |
| Bacillus barbaricus | 0.0055 | 0.00140 |
| Prevotella pallens | 0.0053 | 0.00593 |
| Streptococcus constellatus | 0.0053 | 0.09803 |
| Acinetobacter calcoaceticus | 0.0051 | 0.00000 |
| Fusobacterium necrophorum | 0.0051 | 0.00784 |
| Vagococcus fessus | 0.0050 | 0.01335 |
| Enterococcus faecalis | 0.0050 | 0.00114 |
| Neisseria elongata | 0.0048 | 1.40409 |
| Clostridium bolteae | 0.0047 | 0.00120 |
| Klebsiella pneumoniae | 0.0046 | 0.00035 |
| Aquabacterium commune | 0.0046 | 0.00278 |
| Nostocoida limicola | 0.0044 | 0.00733 |
| Bacteroides caccae | 0.0044 | 0.00143 |
| Arthrobacter crystallopoietes | 0.0043 | 0.00108 |
| Sneathia sanguinegens | 0.0043 | 0.08524 |
| Lactobacillus oris | 0.0041 | 0.00016 |
| Aeromonas veronii | 0.0041 | 0.00055 |
| Acinetobacter haemolyticus | 0.0041 | 0.00000 |
| Aneurinibacillus danicus | 0.0040 | 0.00296 |
| Eubacterium sulci | 0.0038 | 0.06135 |
| Acinetobacter radioresistens | 0.0037 | 0.00000 |
| Phocoenobacter uteri | 0.0036 | 0.00376 |
| Kocuria polaris | 0.0036 | 0.00018 |
| Viridibacillus arenosi | 0.0035 | 0.00035 |
| Empedobacter brevis | 0.0035 | 0.00100 |
| Staphylococcus sciuri | 0.0034 | 0.00000 |
| Aeromonas salmonicida | 0.0033 | 0.00000 |
| Acinetobacter ursingii | 0.0033 | 0.00000 |
| Chitinibacter tainanensis | 0.0033 | 0.00211 |
| Uruburuella suis | 0.0033 | 0.00632 |
| Streptococcus gallolyticus | 0.0032 | 0.01962 |
| Clostridium algidixylanolyticum | 0.0032 | 0.00569 |
| Clostridium colinum | 0.0031 | 0.01366 |
| Corynebacterium tuberculostearicum | 0.0031 | 0.00055 |
| Ammoniphilus oxalivorans | 0.0030 | 0.00228 |
| Intrasporangium calvum | 0.0028 | 0.00053 |
| Bifidobacterium adolescentis | 0.0028 | 0.00104 |
| Brevibacterium samyangensis | 0.0025 | 0.00064 |
| Geobacillus kaustophilus | 0.0025 | 0.00000 |
| Clostridium thermosuccinogenes | 0.0025 | 0.00560 |
| Atopobium rimae | 0.0025 | 0.12052 |
| Erwinia persicina | 0.0024 | 0.00000 |
| Enterococcus asini | 0.0024 | 0.00053 |
| Thermus thermophilus | 0.0024 | 0.00000 |
| Arthrobacter russicus | 0.0024 | 0.00000 |
| Acinetobacter junii | 0.0024 | 0.00083 |
| Corynebacterium argentoratense | 0.0024 | 0.00033 |
| Actinobacillus capsulatus | 0.0023 | 0.00119 |
| Porphyromonas gingivalis | 0.0022 | 0.00110 |
| Vibrio parahaemolyticus | 0.0022 | 0.00872 |
| Pectobacterium carotovorum | 0.0022 | 0.00279 |
| Arthrobacter atrocyaneus | 0.0022 | 0.00156 |
| Candidatus Amoebinatus | 0.0022 | 0.00000 |
| Nitrincola lacisaponensis | 0.0020 | 0.00387 |
| Lactobacillus sakei | 0.0020 | 0.00148 |
| Selenomonas flueggei-like | 0.0019 | 0.03488 |
| Pseudoxanthomonas johnstonii | 0.0019 | 0.00000 |
| Chryseobacterium piscium | 0.0019 | 0.00018 |
| Salinivibrio costicola | 0.0019 | 0.00314 |
| Fusobacterium ulcerans | 0.0019 | 0.00016 |
| Comamonas terrigena | 0.0019 | 0.00018 |
| Diaphorobacter nitroreducens | 0.0019 | 0.00071 |
| Streptophyta uncultured | 0.0018 | 0.01224 |
| Bacillus coahuilensis | 0.0018 | 0.00018 |
| Actinomyces canis | 0.0018 | 0.00601 |
| Alvinella pompejana | 0.0018 | 0.00000 |
| Paenibacillus agarexedens | 0.0018 | 0.00068 |
| Cedecea davisae | 0.0018 | 0.00395 |
| Bacteroides acidifaciens | 0.0018 | 0.00063 |
| Nesterenkonia aethiopica | 0.0018 | 0.00097 |
| Bacillus marisflavi | 0.0017 | 0.00035 |
| Neisseria gonorrhoeae | 0.0017 | 0.02655 |
| Serratia rubidaea | 0.0017 | 0.00450 |
| Escherichia coli | 0.0017 | 0.00000 |
| Prevotella marshii | 0.0016 | 0.00000 |
| Streptococcus alactolyticus | 0.0016 | 0.00053 |
| Vitreoscilla stercoraria | 0.0016 | 0.00125 |
| Bacillus arseniciselenatis | 0.0016 | 0.00185 |
| Kurthia gibsonii | 0.0015 | 0.00018 |
| Allobaculum stercoricanis | 0.0015 | 0.00108 |
| Erwinia toletana | 0.0015 | 0.00000 |
| Micrococcus lylae | 0.0015 | 0.00049 |
| Staphylococcus saprophyticus | 0.0014 | 0.00018 |
| Prevotella bryantii | 0.0014 | 0.00018 |
| Candidatus Nostocoida | 0.0014 | 0.00018 |
| Salmonella enterica | 0.0014 | 0.00000 |
| Prevotella bergensis | 0.0013 | 0.03470 |
| Enterococcus faecium | 0.0013 | 0.00130 |
| Ruminococcus obeum | 0.0013 | 0.00313 |
| Megasphaera paucivorans | 0.0013 | 0.00065 |
| Pediococcus dextrinicus | 0.0013 | 0.00185 |
| Arthrobacter agilis | 0.0013 | 0.00751 |
| Bacteroides stercoris | 0.0013 | 0.00000 |
| Kocuria kristinae | 0.0013 | 0.00033 |
| Micrococcus luteus | 0.0013 | 0.00123 |
| Roseburia intestinalis | 0.0012 | 0.02399 |
| Bacillus weihenstephanensis | 0.0012 | 0.00018 |
| Kocuria aegyptia | 0.0012 | 0.00000 |
| Clostridium aminovalericum | 0.0012 | 0.01820 |
| Enterococcus cecorum | 0.0012 | 0.00070 |
| Streptococcus porcinus | 0.0012 | 0.00189 |
| Selenomonas noxia | 0.0012 | 0.26358 |
| Corynebacterium amycolatum | 0.0012 | 0.00049 |
| Bacillus thuringiensis | 0.0011 | 0.00035 |
| Dietzia maris | 0.0011 | 0.00000 |
| Niastella koreensis | 0.0011 | 0.00016 |
| Capnocytophaga ochracea | 0.0011 | 0.00588 |
| Sejongia jeonii | 0.0011 | 0.00454 |
| Nicoletella semolina | 0.0011 | 0.00201 |
| Sporomusa aerivorans | 0.0011 | 0.00200 |
| Mitsuokella multacida | 0.0010 | 0.00243 |
| Staphylococcus aureus | 0.0010 | 0.00018 |
| Prevotella tannerae | 0.0010 | 0.00840 |
| Streptococcus equi | 0.0010 | 0.00100 |
| Enterococcus ratti | 0.0010 | 0.00131 |
| Conchiformibius steedae | 0.0009 | 0.02955 |
| Pasteurella pneumotropica | 0.0009 | 0.00303 |
| Hahella ganghwensis | 0.0009 | 0.00108 |
| Neisseria polysaccharea | 0.0009 | 0.01371 |
| Aeromonas hydrophila | 0.0009 | 0.00088 |
| Desemzia incerta | 0.0009 | 0.00035 |
| Nesterenkonia lutea | 0.0009 | 0.00053 |
| Bacillus macyae | 0.0009 | 0.00000 |
| Bacteroides eggerthii | 0.0009 | 0.00016 |
| Buttiauxella noackiae | 0.0009 | 0.00000 |
| Bifidobacterium saeculare | 0.0009 | 0.00220 |
| Bradyrhizobium japonicum | 0.0009 | 0.00000 |
| Yaniella halotolerans | 0.0009 | 0.00000 |
| Dermabacter hominis | 0.0009 | 0.00048 |
| Propionibacterium granulosum | 0.0008 | 0.00000 |
| Lactobacillus versmoldensis | 0.0008 | 0.00083 |
| Tetragenococcus halophilus | 0.0008 | 0.00080 |
| Morganella morganii | 0.0008 | 0.00000 |
| Candidatus Rhizobium | 0.0008 | 0.00016 |
| Acinetobacter parvus | 0.0008 | 0.00000 |
| Eubacterium limosum | 0.0008 | 0.00078 |
| Ferrimonas marina | 0.0008 | 0.00895 |
| Clostridium hylemonae | 0.0008 | 0.00016 |
| Sporobacter termitidis | 0.0008 | 0.00066 |
| Staphylococcus warneri | 0.0008 | 0.00000 |
| Arcanobacterium hippocoleae | 0.0008 | 0.00630 |
| Bacillus mannanilyticus | 0.0008 | 0.00000 |
| Carnobacterium pleistocenium | 0.0008 | 0.00165 |
| Aggregatibacter aphrophilus | 0.0007 | 0.03019 |
| Arthrobacter nasiphocae | 0.0007 | 0.00035 |
| Enterococcus aquimarinus | 0.0007 | 0.00156 |
| Demetria terragena | 0.0007 | 0.00000 |
| Kocuria marina | 0.0007 | 0.00035 |
| Arthrobacter scleromae | 0.0007 | 0.00000 |
| Acetobacterium carbinolicum | 0.0007 | 0.00064 |
| Carnobacterium funditum | 0.0007 | 0.00099 |
| Porphyromonas macacae | 0.0007 | 0.00000 |
| Corynebacterium felinum | 0.0007 | 0.00000 |
| Candidatus Chryseobacterium | 0.0006 | 0.00000 |
| Propionispora vibrioides | 0.0006 | 0.00086 |
| Schwartzia succinivorans | 0.0006 | 0.17036 |
| Corynebacterium camporealensis | 0.0006 | 0.00018 |
| Serratia liquefaciens | 0.0006 | 0.00000 |
| Bacillus aquimaris | 0.0006 | 0.00000 |
| Ornithinimicrobium humiphilum | 0.0006 | 0.00033 |
| Kingella oralis | 0.0006 | 0.13302 |
| Micropruina glycogenica | 0.0006 | 0.00000 |
| Sebaldella termitidis | 0.0006 | 0.00000 |
| Pseudomonas costantinii | 0.0006 | 0.00000 |
| Desulfitobacterium hafniense | 0.0006 | 0.00018 |
| Prevotella oris | 0.0006 | 0.02238 |
| Actinomyces hordeovulneris | 0.0006 | 0.00000 |
| Methylobacterium radiotolerans | 0.0006 | 0.00000 |
| Novosphingobium aromaticivorans | 0.0006 | 0.00000 |
| Thermolithobacter sp | 0.0006 | 0.00053 |
| Cronobacter dublinensis | 0.0006 | 0.00000 |
| Abiotrophia defectiva | 0.0005 | 0.00241 |
| Carnobacterium viridans | 0.0005 | 0.00016 |
| Corynebacterium aurimucosum | 0.0005 | 0.00035 |
| Aerococcus urinaehominis | 0.0005 | 0.00121 |
| Clostridium lactatifermentans | 0.0005 | 0.00290 |
| Bacillus jeotgali | 0.0005 | 0.00000 |
| Mitsuokella jalaludinii | 0.0005 | 0.00834 |
| Shewanella aquimarina | 0.0005 | 0.00493 |
| Lachnobacterium bovis | 0.0005 | 0.00051 |
| Achromobacter xylosoxidans | 0.0005 | 0.00137 |
| Bacillus muralis | 0.0005 | 0.00018 |
| Anoxybacillus flavithermus | 0.0005 | 0.00000 |
| Corynebacterium appendicis | 0.0005 | 0.00000 |
| Knoellia sinensis | 0.0005 | 0.00099 |
| Streptococcus hyointestinalis | 0.0005 | 0.00181 |
| Bifidobacterium pseudocatenulatum | 0.0005 | 0.00016 |
| Actinomyces vaccimaxillae | 0.0005 | 0.00018 |
| Enterococcus villorum | 0.0005 | 0.00000 |
| Buttiauxella warmboldiae | 0.0005 | 0.00000 |
| Corynebacterium accolens | 0.0005 | 0.00000 |
| Shewanella baltica | 0.0005 | 0.00000 |
| Shewanella loihica | 0.0005 | 0.00258 |
| Actinobacillus scotiae | 0.0005 | 0.00071 |
| Dialister pneumosintes | 0.0005 | 0.19760 |
| Pseudomonas mosselii | 0.0005 | 0.00000 |
| Mannheimia haemolytica | 0.0005 | 0.00583 |
| Sphingomonas wittichii | 0.0005 | 0.00000 |
| Brevundimonas nasdae | 0.0005 | 0.00000 |
| Pseudomonas meridiana | 0.0005 | 0.00000 |
| Anaerococcus octavius | 0.0005 | 0.00000 |
| Clostridium rectum | 0.0005 | 0.00000 |
| Kocuria varians | 0.0005 | 0.00018 |
| Streptococcus minor | 0.0005 | 0.00018 |
| Clostridium aldrichii | 0.0004 | 0.00064 |
| Acetivibrio cellulolyticus | 0.0004 | 0.00033 |
| Marinospirillum minutulum | 0.0004 | 0.00000 |
| Rhodoferax ferrireducens | 0.0004 | 0.00018 |
| Salana multivorans | 0.0004 | 0.00048 |
| Brachybacterium nesterenkovii | 0.0004 | 0.00066 |
| Chryseobacterium taiwanense | 0.0004 | 0.00000 |
| Streptococcus castoreus | 0.0004 | 0.00033 |
| Deinococcus radiophilus | 0.0004 | 0.00000 |
| Nesterenkonia halobia | 0.0004 | 0.00000 |
| Microbacterium hydrocarbonoxydans | 0.0004 | 0.00000 |
| Kineococcus radiotolerans | 0.0004 | 0.00000 |
| Shigella flexneri | 0.0004 | 0.00000 |
| Bacillus acidicola | 0.0004 | 0.00000 |
| Bacillus cohnii | 0.0004 | 0.00000 |
| Eremococcus coleocola | 0.0004 | 0.00088 |
| Corynebacterium variabile | 0.0004 | 0.00053 |
| Clostridium jejuense | 0.0004 | 0.00254 |
| Bacillus fumarioli | 0.0004 | 0.00000 |
| Bacteroides finegoldii | 0.0004 | 0.00000 |
| Terracoccus luteus | 0.0004 | 0.00018 |
| Streptococcus uberis | 0.0004 | 0.00051 |
| Pseudoxanthomonas kaohsiungensis | 0.0004 | 0.00000 |
| Fusobacterium perfoetens | 0.0004 | 0.00000 |
| Janibacter anophelis | 0.0004 | 0.00000 |
| Paracoccus zeaxanthinifaciens | 0.0004 | 0.00000 |
| Dorea formicigenerans | 0.0004 | 0.00000 |
| Ruminococcus bromii | 0.0004 | 0.00018 |
| Paenibacillus cookii | 0.0004 | 0.00000 |
| Rubrobacter xylanophilus | 0.0004 | 0.00000 |
| Lactobacillus crispatus | 0.0004 | 0.00131 |
| Deinococcus hopiensis | 0.0004 | 0.00000 |
| Bacillus cereus | 0.0004 | 0.00000 |
| Trichococcus flocculiformis | 0.0004 | 0.00000 |
| Bacillus firmus | 0.0003 | 0.00000 |
| Streptococcus intermedius | 0.0003 | 0.02431 |
| Bacteroides nordii | 0.0003 | 0.00000 |
| Selenomonas sputigena | 0.0003 | 0.30165 |
| Paenibacillus azoreducens | 0.0003 | 0.00000 |
| Vagococcus fluvialis | 0.0003 | 0.00000 |
| Actinomyces radicidentis | 0.0003 | 0.00432 |
| Bacillus sporothermodurans | 0.0003 | 0.00055 |
| Stenotrophomonas maltophilia | 0.0003 | 0.00000 |
| Rhodobacter blasticus | 0.0003 | 0.00000 |
| Marinospirillum alkaliphilum | 0.0003 | 0.00000 |
| Aerococcus sanguinicola | 0.0003 | 0.00134 |
| Prevotella disiens | 0.0003 | 0.00086 |
| Staphylococcus equorum | 0.0003 | 0.00000 |
| Tannerella forsythensis | 0.0003 | 0.00558 |
| Shewanella japonica | 0.0003 | 0.00337 |
| Ferrania halotolerans | 0.0003 | 0.00066 |
| Pseudoxanthomonas mexicana | 0.0003 | 0.00000 |
| Clostridium aldenense | 0.0003 | 0.00053 |
| Haemophilus paraphrohaemolyticus | 0.0003 | 0.00116 |
| Actinoplanes capillaceus | 0.0003 | 0.00018 |
| Fusobacterium mortiferum | 0.0003 | 0.00000 |
| Eubacterium infirmum | 0.0003 | 0.01598 |
| Kocuria rosea | 0.0003 | 0.00016 |
| Corynebacterium jeikeium | 0.0003 | 0.00000 |
| Pseudomonas extremorientalis | 0.0003 | 0.00000 |
| Shewanella decolorationis | 0.0003 | 0.00016 |
| Saccharococcus thermophilus | 0.0003 | 0.00000 |
| Olavius crassitunicatus | 0.0003 | 0.00000 |
| Pasteurella langaaensis | 0.0003 | 0.00000 |
| Leminorella grimontii | 0.0003 | 0.00000 |
| Arthrobacter aurescens | 0.0003 | 0.00152 |
| Pasteurella multocida | 0.0003 | 0.00246 |
| Gemella bergeri | 0.0003 | 0.00066 |
| Anaerofustis stercorihominis | 0.0003 | 0.00242 |
| Clostridium methoxybenzovorans | 0.0003 | 0.00000 |
| Chryseobacterium wanjuense | 0.0003 | 0.00385 |
| Aneurinibacillus terranovensis | 0.0003 | 0.00035 |
| Bacteroides fragilis | 0.0003 | 0.00016 |
| Lactococcus raffinolactis | 0.0003 | 0.00000 |
| Prevotella enoeca | 0.0003 | 0.01346 |
| Oceanobacillus oncorhynchi | 0.0003 | 0.00000 |
| Bifidobacterium bifidum | 0.0003 | 0.00094 |
| Paenibacillus campinasensis | 0.0003 | 0.00016 |
| Eubacterium eligens | 0.0003 | 0.00135 |
| Jonesia quinghaiensis | 0.0003 | 0.00000 |
| Lactobacillus gasseri | 0.0002 | 0.02364 |
| Lactobacillus homohiochii | 0.0002 | 0.00000 |
| Enterococcus sulfureus | 0.0002 | 0.00000 |
| Bergeyella zoohelcum | 0.0002 | 0.00018 |
| Sphingomonas aurantiaca | 0.0002 | 0.00000 |
| Chryseobacterium scophthalmum | 0.0002 | 0.00000 |
| Eubacterium ruminantium | 0.0002 | 0.00070 |
| Brevundimonas variabilis | 0.0002 | 0.00000 |
| Pseudochrobactrum asaccharolyticum | 0.0002 | 0.00000 |
| Staphylococcus lentus | 0.0002 | 0.00000 |
| Prevotella albensis | 0.0002 | 0.01166 |
| Algoriphagus mannitolivorans | 0.0002 | 0.00000 |
| Acidaminobacter hydrogenoformans | 0.0002 | 0.00000 |
| Propionispora hippei | 0.0002 | 0.00086 |
| Geobacillus thermodenitrificans | 0.0002 | 0.00000 |
| Candidatus Prevotella | 0.0002 | 0.00515 |
| Thermincola carboxydiphila | 0.0002 | 0.00035 |
| Planomonospora sphaerica | 0.0002 | 0.00000 |
| Geobacillus subterraneus | 0.0002 | 0.00000 |
| Quadrisphaera granulorum | 0.0002 | 0.00066 |
| Hespellia porcina | 0.0002 | 0.01480 |
| Enterococcus casseliflavus | 0.0002 | 0.00000 |
| Tissierella praeacuta | 0.0002 | 0.01391 |
| Streptococcus iniae | 0.0002 | 0.00152 |
| Sporobacterium olearium | 0.0002 | 0.00103 |
| Bacillus aeolius | 0.0002 | 0.00000 |
| Raoultella terrigena | 0.0002 | 0.00000 |
| Sporomusa rhizae | 0.0002 | 0.00033 |
| Fusibacter paucivorans | 0.0002 | 0.00187 |
| Parasporobacterium paucivorans | 0.0002 | 0.00033 |
| Enterococcus pallens | 0.0002 | 0.00000 |
| Enterococcus durans | 0.0002 | 0.00000 |
| Bacillus silvestris | 0.0002 | 0.00000 |
| Parvimonas micra | 0.0002 | 0.42742 |
| Brevibacillus invocatus | 0.0002 | 0.00000 |
| Fusobacterium necrogenes | 0.0002 | 0.00016 |
| Clostridium felsineum | 0.0002 | 0.00000 |
| Raoultella planticola | 0.0002 | 0.00000 |
| Brevibacillus borstelensis | 0.0002 | 0.00000 |
| Corynebacterium pseudotuberculosis | 0.0002 | 0.00103 |
| Cyanobacteria unclassified | 0.0002 | 0.01457 |
| Edwardsiella hoshinae | 0.0002 | 0.00000 |
| Actinobacillus equuli | 0.0002 | 0.00983 |
| Novosphingobium subterraneum | 0.0002 | 0.00000 |
| Alkaliphilus metalliredigens | 0.0002 | 0.00018 |
| Methylobacterium fujisawaense | 0.0002 | 0.00073 |
| Curvibacter delicatus | 0.0002 | 0.00016 |
| Prevotella intermedia | 0.0002 | 0.03262 |
| Gemmatimonas aurantiaca | 0.0002 | 0.00000 |
| Fusobacterium gonidiaformans | 0.0002 | 0.00018 |
| Mannheimia varigena | 0.0002 | 0.02181 |
| Hydrocarboniphaga effusa | 0.0002 | 0.00000 |
| Campylobacter hominis | 0.0002 | 0.01272 |
| Hymenobacter aerophilus | 0.0002 | 0.00000 |
| Rhizobium huautlense | 0.0002 | 0.00000 |
| Brachybacterium sacelli | 0.0002 | 0.00000 |
| Pseudomonas chlororaphis | 0.0002 | 0.00000 |
| Weissella cibaria | 0.0002 | 0.00018 |
| Streptococcus pyogenes | 0.0002 | 0.00528 |
| Leptothrix discophora | 0.0002 | 0.00000 |
| Enterobacter gergoviae | 0.0002 | 0.00000 |
| Lentzea albidocapillata | 0.0002 | 0.00048 |
| Corynebacterium coyleae | 0.0002 | 0.00000 |
| Staphylococcus auricularis | 0.0002 | 0.00000 |
| Dyadobacter fermentans | 0.0002 | 0.00000 |
| Anaerovorax odorimutans | 0.0002 | 0.01360 |
| Alkanindiges illinoisensis | 0.0002 | 0.00000 |
| Psychrobacter cryohalolentis | 0.0002 | 0.00000 |
| Corynebacterium confusum | 0.0002 | 0.00000 |
| Corynebacterium kutscheri | 0.0002 | 0.00068 |
| Capnocytophaga sputigena | 0.0002 | 0.00153 |
| Fusobacterium simiae | 0.0002 | 0.00210 |
| Syntrophococcus sucromutans | 0.0002 | 0.00350 |
| Mannheimia granulomatis | 0.0002 | 0.02420 |
| Anaerovibrio lipolyticus | 0.0002 | 0.00199 |
| Dorea longicatena | 0.0002 | 0.00000 |
| Coprococcus catus | 0.0002 | 0.00000 |
| Kytococcus sedentarius | 0.0002 | 0.00000 |
| Clostridium fimetarium | 0.0002 | 0.00018 |
| Brevundimonas intermedia | 0.0002 | 0.00000 |
| Rhizobium daejeonense | 0.0002 | 0.00018 |
| Bathymodiolus brevior | 0.0002 | 0.00000 |
| Sphingomonas yunnanensis | 0.0002 | 0.00000 |
| Enterobacter hormaechei | 0.0002 | 0.00000 |
| Clostridium methylpentosum | 0.0002 | 0.00018 |
| Weeksella virosa | 0.0002 | 0.00000 |
| Microvirga subterranea | 0.0002 | 0.00000 |
| Flexibacter flexilis | 0.0002 | 0.00000 |
| Actinoplanes derwentensis | 0.0002 | 0.00000 |
| Yaniella flava | 0.0002 | 0.00016 |
| Staphylococcus carnosus | 0.0002 | 0.00000 |
| Clostridium viride | 0.0002 | 0.00033 |
| Sphingobacterium thalpophilum | 0.0002 | 0.00000 |
| Marinobacterium georgiense | 0.0002 | 0.00000 |
| Planomicrobium psychrophilum | 0.0002 | 0.00000 |
| Paracoccus koreensis | 0.0002 | 0.00000 |
| Peptococcus niger | 0.0002 | 0.02719 |
| Jeotgalicoccus pinnipedialis | 0.0002 | 0.00000 |
| Nostoc sp. PCC 8976 | 0.0002 | 0.00000 |
| Bacillus infernus | 0.0002 | 0.00000 |
| Cohnella thermotolerans | 0.0002 | 0.00000 |
| Enterococcus phoeniculicola | 0.0002 | 0.00018 |
| Campylobacter fetus | 0.0002 | 0.00068 |
| Clostridium straminisolvens | 0.0002 | 0.00070 |
| Serratia ureilytica | 0.0002 | 0.00000 |
| Burkholderia terrae | 0.0002 | 0.00000 |
| Propionibacterium propionicum | 0.0002 | 0.00018 |
| Campylobacter showae | 0.0002 | 0.02066 |
| Holdemania filiformis | 0.0002 | 0.00202 |
| Gigantidas gladius | 0.0002 | 0.00018 |
| Moorella thermoacetica | 0.0002 | 0.00016 |
| Lactobacillus acidipiscis | 0.0002 | 0.00053 |
| Anaerostipes caccae | 0.0002 | 0.00121 |
| Alicyclobacillus acidiphilus | 0.0002 | 0.00035 |
| Clostridium novyi | 0.0002 | 0.00000 |
| Epilithonimonas tenax | 0.0002 | 0.00000 |
| Azohydromonas australica | 0.0002 | 0.00000 |
| Ammoniphilus oxalaticus | 0.0002 | 0.00066 |
| Syntrophomonas erecta | 0.0002 | 0.00000 |
| Mitsuaria chitosanitabida | 0.0002 | 0.00000 |
| Belnapia moabensis | 0.0002 | 0.00000 |
| Methylobacterium hispanicum | 0.0002 | 0.00000 |
| Kurthia sibirica | 0.0002 | 0.00018 |
| Staphylococcus pasteuri | 0.0002 | 0.00000 |
| Clostridium scindens | 0.0002 | 0.00049 |
| Actinobacillus succinogenes | 0.0002 | 0.00593 |
| Pelotomaculum thermopropionicum | 0.0002 | 0.00000 |
| Aeromonas enteropelogenes | 0.0002 | 0.00000 |
| Corynebacterium halotolerans | 0.0002 | 0.00000 |
| Marinospirillum insulare | 0.0002 | 0.00000 |
| Blastococcus aggregatus | 0.0002 | 0.00000 |
| Brevibacillus limnophilus | 0.0002 | 0.00175 |
| Facklamia miroungae | 0.0002 | 0.00000 |
| Paracoccus yeei | 0.0002 | 0.00000 |
| Hahella chejuensis | 0.0002 | 0.00356 |
| Turicibacter sanguinis | 0.0002 | 0.00293 |
| Brevibacterium otitidis | 0.0002 | 0.00000 |
| Treponema amylovorum | 0.0002 | 0.55182 |
| Dyadobacter ginsengisoli | 0.0002 | 0.00000 |
| Candidatus Serratia | 0.0002 | 0.00047 |
| Thioflavicoccus mobilis | 0.0002 | 0.00018 |
| Erwinia rhapontici | 0.0002 | 0.00000 |
| Dialister micraerophilus | 0.0001 | 0.00777 |
| Aquabacterium parvum | 0.0001 | 0.00073 |
| Allomonas enterica | 0.0001 | 0.00000 |
| Exiguobacterium aestuarii | 0.0001 | 0.00000 |
| Syntrophomonas wolfei | 0.0001 | 0.00053 |
| Bacillus horikoshii | 0.0001 | 0.00000 |
| Kocuria himachalensis | 0.0001 | 0.00000 |
| Arcicella aquatica | 0.0001 | 0.00000 |
| Pseudomonas hibiscicola | 0.0001 | 0.00000 |
| Owenweeksia hongkongensis | 0.0001 | 0.00000 |
| Selenomonas infelix | 0.0001 | 0.16944 |
| Vibrio halioticoli | 0.0001 | 0.00000 |
| Pseudoxanthomonas daejeonensis | 0.0001 | 0.00000 |
| Zymophilus paucivorans | 0.0001 | 0.00000 |
| Atopobium fossor | 0.0001 | 0.00000 |
| Bifidobacterium merycicum | 0.0001 | 0.00016 |
| Lactobacillus jensenii | 0.0001 | 0.00095 |
| Escherichia albertii | 0.0001 | 0.00000 |
| Bacillus litoralis | 0.0001 | 0.00018 |
| Thermocrispum agreste | 0.0001 | 0.00000 |
| Oceanobacillus iheyensis | 0.0001 | 0.00000 |
| Kocuria carniphila | 0.0001 | 0.00000 |
| Herbaspirillum hiltneri | 0.0001 | 0.17181 |
| Proteus vulgaris | 0.0001 | 0.00000 |
| Eubacterium rectale | 0.0001 | 0.00000 |
| Lactobacillus aviarius | 0.0001 | 0.00000 |
| Clostridium clostridioforme | 0.0001 | 0.00035 |
| Streptomyces libani | 0.0001 | 0.00000 |
| Streptococcus mutans | 0.0001 | 0.00427 |
| Enterococcus avium | 0.0001 | 0.00064 |
| Photobacterium ganghwense | 0.0001 | 0.00033 |
| Lactobacillus kalixensis | 0.0001 | 0.00000 |
| Pseudomonas geniculata | 0.0001 | 0.00000 |
| Pseudomonas monteilii | 0.0001 | 0.00000 |
| Candidatus Endobugula | 0.0001 | 0.00000 |
| Comamonas testosteroni | 0.0001 | 0.00000 |
| Corynebacterium urealyticum | 0.0001 | 0.00000 |
| Anaerosinus glycerini | 0.0001 | 0.00203 |
| Candidatus Contubernalis | 0.0001 | 0.00000 |
| Streptococcus didelphis | 0.0001 | 0.00000 |
| Arthrobacter arilaitensis | 0.0001 | 0.00018 |
| Streptococcus ovis | 0.0001 | 0.00033 |
| Brevibacillus thermoruber | 0.0001 | 0.00018 |
| Curtobacterium flaccumfaciens | 0.0001 | 0.00018 |
| Salinibacillus kushneri | 0.0001 | 0.00000 |
| Pseudoxanthomonas suwonensis | 0.0001 | 0.00000 |
| Microbacterium hominis | 0.0001 | 0.00000 |
| Bacillus soli | 0.0001 | 0.00000 |
| Streptomyces thermoviolaceus | 0.0001 | 0.00000 |
| Corynebacterium simulans | 0.0001 | 0.00000 |
| Glaciecola polaris | 0.0001 | 0.00000 |
| Chryseobacterium formosense | 0.0001 | 0.00048 |
| Bacteroides salyersiae | 0.0001 | 0.00048 |
| Lentzea flaviverrucosa | 0.0001 | 0.00000 |
| Thermus aquaticus | 0.0001 | 0.00000 |
| Promicromonospora vindobonensis | 0.0001 | 0.00000 |
| Rarobacter incanus | 0.0001 | 0.00016 |
| Eubacterium minutum | 0.0000 | 0.00172 |
| Tetrasphaera elongata | 0.0000 | 0.00018 |
| Prevotella zoogleoformans | 0.0000 | 0.00018 |
| Bifidobacterium minimum | 0.0000 | 0.00716 |
| Herbaspirillum frisingense | 0.0000 | 0.00035 |
| Clostridium caminithermale | 0.0000 | 0.00016 |
| Thermoanaerobacter italicus | 0.0000 | 0.00049 |
| Sporosarcina globispora | 0.0000 | 0.00018 |
| Actinomyces slackii | 0.0000 | 0.00063 |
| Simplicispira psychrophila | 0.0000 | 0.00018 |
| Azoarcus buckelii | 0.0000 | 0.00035 |
| Corynebacterium imitans | 0.0000 | 0.00018 |
| Clostridium bifermentans | 0.0000 | 0.00105 |
| Actinomyces gerencseriae | 0.0000 | 0.00587 |
| Novosphingobium pentaromativorans | 0.0000 | 0.00018 |
| Mycoplasma equirhinis | 0.0000 | 0.00018 |
| Bifidobacterium subtile | 0.0000 | 0.00033 |
| Porphyromonas uenonis | 0.0000 | 0.00070 |
| Lactobacillus mucosae | 0.0000 | 0.00016 |
| Sporanaerobacter acetigenes | 0.0000 | 0.00033 |
| Actinomyces urogenitalis | 0.0000 | 0.00127 |
| Acholeplasma vituli | 0.0000 | 0.00018 |
| Clostridium sporogenes | 0.0000 | 0.08149 |
| Hespellia stercorisuis | 0.0000 | 0.00112 |
| Denitratisoma oestradiolicum | 0.0000 | 0.00172 |
| Arthrobacter luteolus | 0.0000 | 0.00018 |
| Actinobacillus minor | 0.0000 | 0.00033 |
| Megamonas hypermegale | 0.0000 | 0.00035 |
| Clostridium populeti | 0.0000 | 0.00070 |
| Pedobacter roseus | 0.0000 | 0.00018 |
| chicken intestinal | 0.0000 | 0.00100 |
| Pontibacter actiniarum | 0.0000 | 0.00088 |
| Escanaba Trough | 0.0000 | 0.00088 |
| Eubacterium uniforme | 0.0000 | 0.00016 |
| Eubacterium brachy | 0.0000 | 0.14905 |
| Lactobacillus kitasatonis | 0.0000 | 0.00596 |
| Desulfovibrio hydrothermalis | 0.0000 | 0.00018 |
| Anaplasma bovis | 0.0000 | 0.00509 |
| Clostridium indolis | 0.0000 | 0.00018 |
| Treponema lecithinolyticum | 0.0000 | 0.06854 |
| Marinilactibacillus psychrotolerans | 0.0000 | 0.00018 |
| Candidatus Arthromitus | 0.0000 | 0.00018 |
| Lactobacillus catenaformis | 0.0000 | 0.00254 |
| Clostridium cylindrosporum | 0.0000 | 0.00088 |
| Lactobacillus frumenti | 0.0000 | 0.00031 |
| Streptococcus anginosus | 0.0000 | 0.00764 |
| Prevotella buccae | 0.0000 | 0.00018 |
| Coriobacterium glomerans | 0.0000 | 0.00016 |
| Geitlerinema carotinosum AICB 37 | 0.0000 | 0.00284 |
| Mycoplasma alkalescens | 0.0000 | 0.00018 |
| Clostridium septicum | 0.0000 | 0.00048 |
| Streptococcus thoraltensis | 0.0000 | 0.00116 |
| Mannheimia ruminalis | 0.0000 | 0.00088 |
| Actinomyces oricola | 0.0000 | 0.00049 |
| Thermovirga lienii | 0.0000 | 0.00948 |
| Arthrobacter mysorens | 0.0000 | 0.00018 |
| Paenibacillus alginolyticus | 0.0000 | 0.00049 |
| Selenomonas dianae | 0.0000 | 0.07091 |
| Facklamia hominis | 0.0000 | 0.00016 |
| Mycoplasma hyosynoviae | 0.0000 | 0.01639 |
| Paenibacillus glycanilyticus | 0.0000 | 0.00066 |
| Capnocytophaga gingivalis | 0.0000 | 0.00119 |
| Clostridium ganghwense | 0.0000 | 0.00018 |
| Cardiobacterium hominis | 0.0000 | 0.01004 |
| Campylobacter insulaenigrae | 0.0000 | 0.00018 |
| Sphingobium yanoikuyae | 0.0000 | 0.00018 |
| Succiniclasticum ruminis | 0.0000 | 0.00018 |
| Dethiosulfovibrio acidaminovorans | 0.0000 | 0.00086 |
| Mogibacterium timidum | 0.0000 | 0.08052 |
| Megasphaera elsdenii | 0.0000 | 0.00016 |
| Campylobacter hyointestinalis | 0.0000 | 0.07403 |
| Asteroleplasma anaerobium | 0.0000 | 0.01996 |
| Brucella melitensis | 0.0000 | 0.00035 |
| Bordetella pertussis | 0.0000 | 0.00018 |
| Mycoplasma faucium | 0.0000 | 0.26901 |
| Clostridium glycolicum | 0.0000 | 0.00018 |
| Weissella soli | 0.0000 | 0.00018 |
| Clostridium peptidivorans | 0.0000 | 0.00035 |
| Acholeplasma modicum | 0.0000 | 0.00053 |
| Prevotella loescheii | 0.0000 | 0.00063 |
| Actinomyces denticolens | 0.0000 | 0.00031 |
| Psychrobacter celer | 0.0000 | 0.00018 |
| Eubacteriaceae oral | 0.0000 | 0.07583 |
| Nitrosomonas oligotropha | 0.0000 | 0.00070 |
| Eubacterium biforme | 0.0000 | 0.00016 |
| Duganella violaceinigra | 0.0000 | 0.00016 |
| Lactobacillus kimchii | 0.0000 | 0.00055 |
| Clostridium grantii | 0.0000 | 0.00035 |
| Spirochaeta stenostrepta | 0.0000 | 0.00018 |
| Bordetella parapertussis | 0.0000 | 0.00275 |
| Actinomyces hongkongensis | 0.0000 | 0.00047 |
| TM7 phylum sp. oral taxon 356 | 0.0000 | 0.06335 |
| Clostridium bartlettii | 0.0000 | 0.00018 |
| Eubacterium barkeri | 0.0000 | 0.00161 |
| Janthinobacterium agaricidamnosum | 0.0000 | 0.00088 |
| Neisseria dentiae | 0.0000 | 0.01497 |
| Glycomyces harbinensis | 0.0000 | 0.00018 |
| Bifidobacterium pullorum | 0.0000 | 0.00803 |
| Anaerobiospirillum succiniciproducens | 0.0000 | 0.00016 |
| Garciella nitratireducens | 0.0000 | 0.00035 |
| Paucimonas lemoignei | 0.0000 | 0.00844 |
| Prevotella nigrescens | 0.0000 | 0.01187 |
| Brevibacterium antiquum | 0.0000 | 0.00018 |
| Papillibacter cinnamivorans | 0.0000 | 0.00018 |
| Butyrivibrio fibrisolvens | 0.0000 | 0.00648 |
| Paenibacillus larvae | 0.0000 | 0.00018 |
| Corynebacterium matruchotii | 0.0000 | 0.10839 |
| Rhizobium leguminosarum | 0.0000 | 0.00051 |
| Gracilibacter thermotolerans | 0.0000 | 0.00263 |
| Shuttleworthia satelles | 0.0000 | 0.04393 |
| Clostridium pascui | 0.0000 | 0.00018 |
| Campylobacter sputorum | 0.0000 | 0.28068 |
| Lactococcus garvieae | 0.0000 | 0.00018 |
| Lachnospira pectinoschiza | 0.0000 | 0.00222 |
| Mycoplasmataceae | 0.0000 | 0.00033 |
| Treponema medium | 0.0000 | 0.86970 |
| Anaeroglobus geminatus | 0.0000 | 0.05631 |
| Olsenella uli | 0.0000 | 0.00210 |
| TM7 phylum sp. oral taxon 348 | 0.0000 | 0.01242 |
| Sporomusa sphaeroides | 0.0000 | 0.00051 |
| Bifidobacterium scardovii | 0.0000 | 0.00033 |
| Porphyromonas circumdentaria | 0.0000 | 0.00018 |
| Prevotella baroniae | 0.0000 | 0.00205 |
| Thermanaerovibrio velox | 0.0000 | 0.00018 |
| Clostridium litorale | 0.0000 | 0.00121 |
| Dethiosulfovibrio peptidovorans | 0.0000 | 0.00016 |
| Lochheadia duodecas | 0.0000 | 0.00080 |
| Clostridium perfringens | 0.0000 | 0.00018 |
| Acetobacterium woodii | 0.0000 | 0.00053 |
| Simplicispira metamorpha | 0.0000 | 0.00018 |
| Renibacterium salmoninarum | 0.0000 | 0.00018 |
| Mycoplasma subdolum | 0.0000 | 0.03729 |
| Tetrathiobacter kashmirensis | 0.0000 | 0.00140 |
| Macrococcus caseolyticus | 0.0000 | 0.00016 |
| Corynebacterium glutamicum | 0.0000 | 0.00135 |
| Anaerococcus prevotii | 0.0000 | 0.00049 |
| Treponema berlinense | 0.0000 | 0.00640 |
| Propionispira arboris | 0.0000 | 0.00051 |
| Eubacterium nodatum | 0.0000 | 0.04084 |
| Desulfonatronovibrio hydrogenovorans | 0.0000 | 0.00018 |
| Corynebacterium durum | 0.0000 | 0.02325 |
| Dialister invisus | 0.0000 | 0.22032 |
| Legionella busanensis | 0.0000 | 0.00018 |
| Acetobacter orleanensis | 0.0000 | 0.00018 |
| Selenomonas lacticifex | 0.0000 | 0.00049 |
| Thiobacter subterraneus | 0.0000 | 0.00016 |
| Halothermothrix orenii | 0.0000 | 0.00018 |
| Alkalibacterium iburiense | 0.0000 | 0.00018 |
| Propioniferax innocua | 0.0000 | 0.00110 |
| Clostridium oroticum | 0.0000 | 0.00018 |
| Clostridium botulinum | 0.0000 | 0.08228 |
| Mogibacterium vescum | 0.0000 | 0.00033 |
| Eubacterium ventriosum | 0.0000 | 0.00138 |
| Campylobacter helveticus | 0.0000 | 0.00033 |
| Herbaspirillum chlorophenolicum | 0.0000 | 0.00088 |
| Filifactor villosus | 0.0000 | 0.81886 |
| Actinobaculum urinale | 0.0000 | 0.00237 |
| Paenibacillus lentimorbus | 0.0000 | 0.00018 |
| Rhodococcus gordoniae | 0.0000 | 0.00018 |
| Castellaniella denitrificans | 0.0000 | 0.00053 |
| Candidatus Glomeribacter | 0.0000 | 0.00070 |
| Variovorax dokdonensis | 0.0000 | 0.00018 |
| Lactobacillus panis | 0.0000 | 0.00286 |
| Atopobium vaginae | 0.0000 | 0.00096 |
| Mycoplasma gateae | 0.0000 | 0.00070 |
| Sulfurivirga caldicularium | 0.0000 | 0.00018 |
| Eubacterium callanderi | 0.0000 | 0.00018 |
| Actinomyces israelii | 0.0000 | 0.00302 |
| Helicobacter trogontum | 0.0000 | 0.00018 |
| Clostridium ultunense | 0.0000 | 0.00070 |
| Clostridium symbiosum | 0.0000 | 0.00331 |
| Ochrobactrum anthropi | 0.0000 | 0.00035 |
| Prevotella multiformis | 0.0000 | 0.01268 |
| Campylobacter jejuni | 0.0000 | 0.00539 |
| Lactobacillus fermentum | 0.0000 | 0.00578 |
| Clostridium stercorarium | 0.0000 | 0.00018 |
| Ehrlichia canis | 0.0000 | 0.00018 |
| Salinibacterium amurskyense | 0.0000 | 0.00070 |
| Porphyromonas asaccharolytica | 0.0000 | 0.00035 |
| Weissella thailandensis | 0.0000 | 0.00018 |
| Prevotella denticola | 0.0000 | 0.00018 |
| Alicyclobacillus pomorum | 0.0000 | 0.00018 |
| Herbaspirillum seropedicae | 0.0000 | 0.04842 |
| Clostridium sphenoides | 0.0000 | 0.00018 |
| Bifidobacterium dentium | 0.0000 | 0.00887 |
| Paenibacillus mendelii | 0.0000 | 0.00033 |
| Eubacterium hallii | 0.0000 | 0.00135 |
| Lonepinella koalarum | 0.0000 | 0.00269 |
| Mycoplasma auris | 0.0000 | 0.04216 |
| Lactobacillus pontis | 0.0000 | 0.00159 |
| Bacillus algicola | 0.0000 | 0.00033 |
| Eubacterium acidaminophilum | 0.0000 | 0.00018 |
| Phaseolus vulgaris | 0.0000 | 0.00031 |
| Capnocytophaga canimorsus | 0.0000 | 0.00018 |
| Marinibacillus campisalis | 0.0000 | 0.00135 |
| Helcococcus sueciensis | 0.0000 | 0.00035 |
| Thermosyntropha lipolytica | 0.0000 | 0.00068 |
| Hydrogenophaga flava | 0.0000 | 0.00018 |
| Actinomyces viscosus | 0.0000 | 0.07497 |
| Clostridium thiosulfatireducens | 0.0000 | 0.00018 |
| Sphingomonas yabuuchiae | 0.0000 | 0.00018 |
| Clostridium subterminale | 0.0000 | 0.11016 |
| Acholeplasma axanthum | 0.0000 | 0.05224 |
| Pasteurella mairii | 0.0000 | 0.00018 |
| Eubacterium pyruvativorans | 0.0000 | 0.00033 |
| Haemophilus haemoglobinophilus | 0.0000 | 0.00835 |
| Thermoanaerobacterium aotearoense | 0.0000 | 0.00035 |
| Treponema socranskii | 0.0000 | 0.14370 |
| Clostridium irregulare | 0.0000 | 0.00049 |
| Gulosibacter molinativorax | 0.0000 | 0.00084 |
| Pectinatus haikarae | 0.0000 | 0.00053 |
| Clostridium orbiscindens | 0.0000 | 0.00018 |
| Prevotella heparinolytica | 0.0000 | 0.00016 |
| Duganella zoogloeoides | 0.0000 | 0.00018 |
| Pectinatus portalensis | 0.0000 | 0.00070 |
| Herbaspirillum lusitanum | 0.0000 | 0.08360 |
| Treponema denticola | 0.0000 | 0.84692 |
| Mycoplasma feliminutum | 0.0000 | 0.09678 |
| Ethanoligenens harbinense | 0.0000 | 0.00035 |
| Eubacterium saphenum | 0.0000 | 0.27413 |
| Butyrivibrio hungatei | 0.0000 | 0.00168 |
| Alkaliphilus oremlandii | 0.0000 | 0.00035 |
| Brachybacterium muris | 0.0000 | 0.00016 |
| Ideonella dechloratans | 0.0000 | 0.00035 |
| Finegoldia magna | 0.0000 | 0.01879 |
| Bacillus pycnus | 0.0000 | 0.00016 |
| Sporacetigenium mesophilum | 0.0000 | 0.00018 |
| Clostridium propionicum | 0.0000 | 0.00070 |
| Candidatus Procabacter | 0.0000 | 0.00018 |
| Devosia riboflavina | 0.0000 | 0.00035 |
| Glycomyces algeriensis | 0.0000 | 0.00018 |
| Lactobacillus intestinalis | 0.0000 | 0.00110 |
| Anaeroplasma abactoclasticum | 0.0000 | 0.00193 |
| Sphingobium amiense | 0.0000 | 0.00035 |
| Desulfovibrio desulfuricans | 0.0000 | 0.00018 |
| Gallibacterium anatis | 0.0000 | 0.00018 |
| Eubacterium cellulosolvens | 0.0000 | 0.00082 |
| Acetobacter pomorum | 0.0000 | 0.00018 |
| Pseudonocardia dioxanivorans | 0.0000 | 0.00018 |
| Corynebacterium sundsvallense | 0.0000 | 0.00018 |
| Wolinella succinogenes | 0.0000 | 0.00368 |
| Mycoplasma canadense | 0.0000 | 0.00035 |
| Treponema porcinum | 0.0000 | 0.00524 |
| Clostridium xylanovorans | 0.0000 | 0.00018 |
| Propionibacterium australiense | 0.0000 | 0.00836 |
| Limnobacter thiooxidans | 0.0000 | 0.00219 |
| Corynebacterium singulare | 0.0000 | 0.00018 |
| Anaplasma phagocytophilum | 0.0000 | 0.00088 |
| Asticcacaulis biprosthecium | 0.0000 | 0.00055 |
| Lactobacillus amylolyticus | 0.0000 | 0.00018 |
| Pseudomonas fragi | 0.0000 | 0.00018 |
| Caminicella sporogenes | 0.0000 | 0.00070 |
| Desulfosporosinus meridiei | 0.0000 | 0.00016 |
| Desulfobulbus mediterraneus | 0.0000 | 0.00031 |
| Lactobacillus concavus | 0.0000 | 0.00055 |
| GpI uncultured | 0.0000 | 0.00033 |
| Mycoplasma cloacale | 0.0000 | 0.00016 |
| Brooklawnia cerclae | 0.0000 | 0.00047 |
| Anaerobaculum mobile | 0.0000 | 0.01736 |
| Tepidimicrobium ferriphilum | 0.0000 | 0.00281 |
| Treponema phagedenis | 0.0000 | 0.02219 |
| candidate division TM7 genomosp. P1 | 0.0000 | 0.00016 |
| Hyphomonas johnsonii | 0.0000 | 0.00018 |
| Megasphaera sueciensis | 0.0000 | 0.00049 |
| Eubacterium aggregans | 0.0000 | 0.01431 |
| Mogibacterium pumilum | 0.0000 | 0.00341 |
| Spirochaeta smaragdinae | 0.0000 | 0.00158 |
| Acetobacterium bakii | 0.0000 | 0.00018 |
| Amycolatopsis thermoflava | 0.0000 | 0.00016 |
| Actinomyces bowdenii | 0.0000 | 0.00033 |
| Bifidobacterium choerinum | 0.0000 | 0.00016 |
| Lactobacillus coleohominis | 0.0000 | 0.00047 |
| Eubacterium tenue | 0.0000 | 0.00033 |
| Cryptobacterium curtum | 0.0000 | 0.00439 |
| Herminiimonas aquatilis | 0.0000 | 0.00125 |
| Lactobacillus acidophilus | 0.0000 | 0.00301 |
| Campylobacter gracilis | 0.0000 | 0.13947 |
| Centipeda periodontii | 0.0000 | 0.02214 |
| Spirochaeta zuelzerae | 0.0000 | 0.00055 |
| Actinomyces catuli | 0.0000 | 0.00368 |
| Acidothermus cellulolyticus | 0.0000 | 0.00016 |
| Clostridium frigidicarnis | 0.0000 | 0.00123 |
| Clostridium aerotolerans | 0.0000 | 0.00018 |
| Peptoniphilus lacrimalis | 0.0000 | 0.00209 |
| Herbaspirillum rubrisubalbicans | 0.0000 | 0.00035 |
| Prevotella dentalis | 0.0000 | 0.00612 |
| Parascardovia denticolens | 0.0000 | 0.02740 |
| Clostridium papyrosolvens | 0.0000 | 0.00140 |
| Clostridium acetobutylicum | 0.0000 | 0.00158 |
| Caryophanon latum | 0.0000 | 0.00035 |
| Treponema bryantii | 0.0000 | 0.00031 |
| Acidovorax defluvii | 0.0000 | 0.00018 |
| Clostridium thermopalmarium | 0.0000 | 0.00016 |
| Pelospora glutarica | 0.0000 | 0.00018 |
| Herbaspirillum huttiense | 0.0000 | 0.00018 |
| Asticcacaulis excentricus | 0.0000 | 0.00220 |
| Clostridium fallax | 0.0000 | 0.00140 |
| Tetragenococcus solitarius | 0.0000 | 0.00018 |
| Zymobacter palmae | 0.0000 | 0.00016 |
| Mycoplasma indiense | 0.0000 | 0.00147 |
| Desulfonatronum thiodismutans | 0.0000 | 0.00066 |
| Mycoplasma arthritidis | 0.0000 | 0.00103 |
| Selenomonas ruminantium | 0.0000 | 0.00311 |
| Treponema brennaborense | 0.0000 | 0.00018 |
| Actinomyces georgiae | 0.0000 | 0.00033 |
| Sinorhizobium americanum | 0.0000 | 0.00035 |
| Luteococcus peritonei | 0.0000 | 0.00055 |
| Streptococcus ferus | 0.0000 | 0.00016 |
| GpX uncultured | 0.0000 | 0.00016 |
| Brevibacterium paucivorans | 0.0000 | 0.00031 |
| Tessaracoccus bendigoensis | 0.0000 | 0.00016 |
| Clostridium phytofermentans | 0.0000 | 0.00051 |
| Dietzia psychralcaliphila | 0.0000 | 0.00018 |
| Streptomyces thermospinosisporus | 0.0000 | 0.00048 |
| Gardnerella vaginalis | 0.0000 | 0.01459 |
| Lactobacillus helveticus | 0.0000 | 0.02151 |
| Mycoplasma falconis | 0.0000 | 0.00105 |
| Bacillus macauensis | 0.0000 | 0.00055 |
| Serratia ficaria | 0.0000 | 0.00031 |
| Porphyromonas endodontalis | 0.0000 | 0.03107 |
| Tindallia californiensis | 0.0000 | 0.00018 |
| Corynebacterium mastitidis | 0.0000 | 0.00018 |
| Dechloromonas aromatica | 0.0000 | 0.00084 |
| Actinomyces naeslundii | 0.0000 | 0.00016 |
| Oleispira antarctica | 0.0000 | 0.00018 |
| Corynebacterium ammoniagenes | 0.0000 | 0.00048 |
| Anaeroarcus burkinensis | 0.0000 | 0.00031 |
| Lactobacillus johnsonii | 0.0000 | 0.00016 |
| Dechloromonas agitata | 0.0000 | 0.00016 |
| Paenibacillus illinoisensis | 0.0000 | 0.00018 |
| Gordonia namibiensis | 0.0000 | 0.00018 |
| Lactobacillus salivarius | 0.0000 | 0.00035 |
| Campylobacter lari | 0.0000 | 0.00338 |
| Actinobaculum massiliae | 0.0000 | 0.02013 |
| Xenophilus azovorans | 0.0000 | 0.00018 |
| Candidatus Ishikawaella | 0.0000 | 0.00055 |
| Eubacterium yurii | 0.0000 | 0.07848 |
| Treponema maltophilum | 0.0000 | 0.08625 |
| Photobacterium phosphoreum | 0.0000 | 0.00031 |
| Cardiobacterium valvarum | 0.0000 | 0.00175 |
| Arthrobacter methylotrophus | 0.0000 | 0.00048 |
| Olsenella profusa | 0.0000 | 0.00173 |
| Heliorestis baculata | 0.0000 | 0.00016 |
| Actinoalloteichus spitiensis | 0.0000 | 0.00048 |
| Bacteroidales oral | 0.0000 | 0.00174 |
| Anaeromusa acidaminophila | 0.0000 | 0.00112 |
| Sporomusa ovata | 0.0000 | 0.00033 |
| Clostridium argentinense | 0.0000 | 0.00105 |
| Treponema putidum | 0.0000 | 0.08079 |
| Bacillus arsenicus | 0.0000 | 0.00018 |
| TM7 phylum sp. oral clone BU080 | 0.0000 | 0.00051 |
| Clostridium schirmacherense | 0.0000 | 0.00228 |
| Syntrophus aciditrophicus | 0.0000 | 0.00018 |
| Geobacter sulfurreducens | 0.0000 | 0.00035 |
| Schlegelella thermodepolymerans | 0.0000 | 0.00053 |
| Eikenella corrodens | 0.0000 | 0.01737 |
| Clostridium difficile | 0.0000 | 0.00312 |
| Thermoanaerobacter pseudethanolicus | 0.0000 | 0.00018 |
| Syntrophomonas sapovorans | 0.0000 | 0.00018 |
| Herminiimonas fonticola | 0.0000 | 0.00332 |
| Arthrobacter ureafaciens | 0.0000 | 0.00018 |
| Actinobacillus ureae | 0.0000 | 0.00018 |
| Clostridium drakei | 0.0000 | 0.00035 |
| Clostridium chauvoei | 0.0000 | 0.00088 |
| Candidatus Peptoniphilus | 0.0000 | 0.00055 |
| Arthrobacter albus | 0.0000 | 0.00018 |
| Actinomyces marimammalium | 0.0000 | 0.00016 |
| Suttonella indologenes | 0.0000 | 0.00016 |
| Pseudanabaena sp. PCC 6802 | 0.0000 | 0.00035 |
| Desulfobulbus rhabdoformis | 0.0000 | 0.00223 |
| Paenibacillus thiaminolyticus | 0.0000 | 0.00018 |
| Pseudanabaena sp. 0tu30s18 | 0.0000 | 0.00016 |
| Lactobacillus amylovorus | 0.0000 | 0.00145 |
| Ehrlichia chaffeensis | 0.0000 | 0.00018 |
| Photobacterium leiognathi | 0.0000 | 0.00018 |
| Legionella-like amoebal | 0.0000 | 0.00018 |
| TM7 phylum sp. oral clone EW055 | 0.0000 | 0.00140 |
| Treponema pectinovorum | 0.0000 | 0.00869 |
| Scardovia inopinata | 0.0000 | 0.19093 |
| Treponema parvum | 0.0000 | 0.01811 |
| Clostridium intestinale | 0.0000 | 0.00018 |
| Cellulomonas denverensis | 0.0000 | 0.00018 |
| Aminobacterium colombiense | 0.0000 | 0.17962 |
| Sporosarcina aquimarina | 0.0000 | 0.00031 |
| Bordetella petrii | 0.0000 | 0.00121 |
| Propionivibrio pelophilus | 0.0000 | 0.00055 |
| Collimonas fungivorans | 0.0000 | 0.00221 |
| Arthrobacter globiformis | 0.0000 | 0.00018 |
| Paludibacter propionicigenes | 0.0000 | 0.00119 |
| Pseudobutyrivibrio ruminis | 0.0000 | 0.00082 |
| Desulfobulbus elongatus | 0.0000 | 0.00256 |
| OP10 genera incertae sedis uncultured | 0.0000 | 0.00283 |
| Clostridium saccharolyticum | 0.0000 | 0.00363 |
| Beggiatoa alba | 0.0000 | 0.00018 |
| Alkaliphilus crotonatoxidans | 0.0000 | 0.00136 |
| Lactobacillus reuteri | 0.0000 | 0.00296 |
| Clostridium putrefaciens | 0.0000 | 0.00018 |
| Capnocytophaga granulosa | 0.0000 | 0.00123 |
| Eubacterium angustum | 0.0000 | 0.00018 |
| Klebsiella singaporensis | 0.0000 | 0.00048 |
| Arthrobacter ardleyensis | 0.0000 | 0.00048 |
| Ruminococcus lactaris | 0.0000 | 0.00033 |
| Rhizobium sullae | 0.0000 | 0.00018 |
| Clostridium aceticum | 0.0000 | 0.00018 |
| Clostridium cellulolyticum | 0.0000 | 0.00018 |
| Desulfomicrobium orale | 0.0000 | 0.00495 |
| Corynebacterium thomssenii | 0.0000 | 0.00051 |
| Enterococcus haemoperoxidus | 0.0000 | 0.00018 |
